# Using a Rapid Knowledge Translation Approach for Better Sexual and Reproductive Health and Rights in Bangladesh, Burundi, Indonesia, and Jordan

**DOI:** 10.9745/GHSP-D-21-00461

**Published:** 2022-04-28

**Authors:** Kimberley Meijers, Anke van der Kwaak, Ibrahim Aqel, Robert Borst, Françoise Jenniskens, Aryanti Radyowijati, Arnob Chakrabarty, Eric Nzeyimana, Ely Sawitri, Noor Tromp

**Affiliations:** a KIT Royal Tropical Institute, Amsterdam, Netherlands.; b Institute for Family Health, King Hussein Foundation, Amman, Jordan.; c Erasmus School of Health Policy & Management, Rotterdam, Netherlands.; d Independent senior sexual and reproductive health and rights expert, Wittem, Netherlands.; e ResultsinHealth, Leiderdorp, Netherlands.; f RedOrange Media and Communications, Bangladesh.; g East African Community, Tanzania.; h Rutgers WPF Indonesia, Indonesia.

## Abstract

**Introduction:**

Translation of knowledge into policy and practice is important to prevent sexual reproductive health and rights (SRHR)-related morbidity and mortality and ensure access to rights. Existing approaches to knowledge translation are often relatively rigid and implicitly assume linear processes, leading to time-consuming processes that are not tailored to countries' needs.

**Approach:**

SRHR knowledge platforms designed and implemented a collaborative rapid improvement model for knowledge translation (CRIM-KT) in Burundi, Bangladesh, Indonesia, and Jordan. The approach consisted of learning sessions and action periods aimed at improving policies and practices addressing the prevention of child marriage and teenage pregnancies. To evaluate the approach, a participatory action learning process took place throughout the implementation (September 2017 and January 2019). An end evaluation was conducted using a desk review of project documentation, in-depth interviews, and a focus group discussion to document the process, outcomes, and lessons learned.

**Achievements:**

In Indonesia, a local government policy was changed that aims to prevent child marriage by avoiding misinterpretation of a local cultural practice. In Jordan, the cabinet endorsed a national action plan to prevent child marriage and changes in practice took place. In Burundi, no tangible changes in policy and practice in SRHR were observed. In Bangladesh, practice changed by strengthening coordination for collaboration and exchange among stakeholders to prevent child marriage. In all countries, the approach considerably strengthened participants' knowledge translation capacities.

**Conclusion:**

The CRIM-KT led to improvements in policy and practice in a relatively short time frame and different contexts. This can be explained by the systematic, structured, and participatory approach, allowing for contextual adaptation and involvement of stakeholders, as well as the cross-learning on 2 levels (international and country collaboration teams). The principles of the CRIM-KT may be further developed and applied in other fields in global health to strengthen knowledge translation processes.

## Abstract (precis)

There is a growing need for approaches to support rapid knowledge translation processes that can create changes in policy and practice and that can apply to different country contexts. The collaborative rapid improvement model for knowledge translation in sexual and reproductive health and rights (SRHR) implemented in 4 countries improved SRHR practice and policies.

## Teaser

**Key Findings:**

- The Collaborative Rapid Improvement Model for Knowledge Translation (CRIM-KT) responds to the growing need in the global health field for methods to support rapid knowledge translation processes that are both structured and flexible, include types of knowledge other than scientific, and that foster dialogue and collaboration among researchers, policy makers, practitioners, and private sector actors.
- Key enabling factors for change included the systematic, structured, and participatory nature of the CRIM-KT model approach, which allowed for contextual adaptation and involvement of stakeholders, and cross-learning at the international and country levels.
- Key disabling factors were the short time frame between learning sessions and limited funds available to develop and organize activities during the local action periods.

**Key Implications:**

- This rapid improvement model should be further developed and tested by addressing disabling factors and applying it beyond the sexual reproductive health and rights (SRHR) field.
- Policy makers are encouraged to stimulate and participate in rapid improvement approaches led by knowledge platforms and use the context-specific and evidence-informed outcomes in SRHR policy making.

Résumé en français à la fin de l'article.

## INTRODUCTION

The coronavirus disease (COVID-19) pandemic illustrates a broader global need for the rapid translation of knowledge into policy and practice.^1^^,^^2^ Policy makers and practitioners need to respond quickly to global health problems and issues despite a paucity of contextualized knowledge.^1^^,^^3^^,^^4^ Knowledge platforms can play an important role in prioritizing relevant issues, mapping knowledge gaps, and developing evidence-based policy and practice.^1^^,^^5^

In this article, we use the Canadian Institutes of Health Research's^6^ definition of knowledge translation:

*a dynamic and iterative process that includes synthesis, dissemination, exchange and ethically sound application of knowledge*.

The field of knowledge translation uses tools and methods that seek to increase the use of evidence in decision making while increasing the consideration of policy rationales in evidence generation.^7^ This is deemed crucial for improving program and policy effectiveness and for understanding and engineering lasting change. The use of knowledge and scaling up best practices through knowledge capture and dissemination alone is not sufficient but also requires investing in an organizational enabling environment and technical skills of stakeholders.^8^

Linkages between research, policy, and practice are often considered to be unidirectional and rigid, with relatively static knowledge flows, sometimes referred to as “research uptake.” Uptake focuses on researchers that disseminate their findings to ministries or other scholars interested in the thematic area.^9^^,^^10^ To achieve social change, it is important to have more rapid approaches and to engage stakeholders in a joint process of constructing the required knowledge.^11^ Stakeholders could be potential knowledge users, who possess important experiential knowledge of the policy and practice environments into which the knowledge has to be translated.^12^ Processes of cocreation within knowledge translation can be facilitated by embedding them in dedicated platforms.^5^^,^^13^

Knowledge platforms bring decision makers, researchers, practitioners, civil society groups, and other stakeholders together to facilitate the process of translating evidence into policy and action. They achieve this by aligning research topics with policy priorities; responding to pressing issues through developing different knowledge products like policy briefs, rapid responses, and evidence summaries; and convening dialogues to guide policy formulation and implementation while considering local and political context.^1^ These platforms can provide context-specific and actionable evidence to enable countries to adapt global solutions to local needs and realities.^1^^,^^2^ Knowledge platforms can facilitate multidisciplinary collaboration and learning across contexts and discourses.^1^^,^^3^^,^^5^ Yet, there remains a lack of understanding about how knowledge translation methods can support cross-platform and intercountry learning,^14^ and there is often little coherence between these methods. This undermines efforts to change practices and policies.^15^^,^^16^ Further assessment is needed on which knowledge translation approaches are effective, focusing on how to sustain changes in policy and practice and how to use such approaches across different contexts and platforms.

Further assessment is needed to determine which knowledge translation approaches are effective.

We report on the application of a new approach, the Collaborative Rapid Improvement Model for Knowledge Translation (CRIM-KT), in the context of sexual and reproductive health and rights (SRHR).

### Implementation Setting

The CRIM-KT approach was implemented in the context of Share-Net International, the Knowledge Platform on SRHR (https://share-netinternational.org/), which is hosted by an independent center of expertise, education, and intercultural cooperation for sustainable development in the Netherlands.^17^ Share-Net International has SRHR knowledge platforms in Bangladesh, Burundi, the Netherlands, and Jordan funded by the Dutch Ministry of Foreign Affairs.^18^ The original setup of these knowledge platforms differed by country. In Bangladesh, the platform^19^ is hosted by an organization that works on social and behavioral change communication and advocacy on SRHR issues.^20^ In Burundi, the knowledge platform^21^ was hosted by an international nongovernmental organization (INGO).^22^ The Dutch knowledge platform^23^ focused on Indonesia through an INGO, which implemented CRIM-KT as part of a 5-year program aimed at reducing child marriage, teenage pregnancy, and female genital mutilation/cutting.^24^ Finally, the Jordanian knowledge platform^25^ is hosted by a semigovernmental organization with the authority to direct national efforts on sustainable development, population issues, and reproductive health.^26^ The country platforms and their network of experts and member organizations harness localized knowledge while combining the strengths of key international actors to promote the development of better policies and practices in SRHR.^15^

Supported by the outcomes of 2 external evaluations^5^^,^^27^ (2017), Share-Net International and the country knowledge platform secretariats decided they wanted to invest in knowledge exchange and learning among the knowledge platforms and focus more on knowledge translation and use. As a response, based on the identified needs, a steering committee member of the Jordanian knowledge platform proposed using the CRIM-KT.

## THE CRIM-KT APPROACH

### Design Phase

The CRIM-KT approach aimed to (1) explore and test strategies for translating knowledge into better SRHR policy and practice using the quality improvement Plan-Do-Study-Act (PDSA) learning cycles and (2) institutionalize knowledge translation activities by strengthening capacity and increasing collaboration and exchange between the country knowledge platforms and among their members. Additionally, the knowledge platform aimed to share experiences and lessons learned across the 4 country knowledge platforms.

The CRIM-KT approach aimed to strengthen capacity and increase collaboration and exchange between country knowledge platforms.

The CRIM-KT approach was built on the collaborative approach “Breakthrough Series” that aims to achieve rapid changes in a short period.^28^ The collaborative approach uses a structured but adaptive learning process that allows different stakeholders to experiment with various strategies through PDSA learning cycles and share the results with others to accelerate learning (Box).^28^ Such cycles are commonly used to strengthen organizational processes. The collaborative approach has been used successfully to improve the quality of care and reduce costs among public health centers worldwide.^28^ Quality improvement in health is closely related to the field of knowledge translation to enhance the use of evidence from research to improve health care policy and practice.^29^ By integrating knowledge translation and quality improvement into 1 approach, the strengths of each field can be leveraged for greater impact.^30^

BOX **Plan-Do-Study-Act (PDSA) Learning Cycle**

**Plan:** During the international learning sessions, the knowledge platform representatives propose and plan change strategies including monitoring measures using “change packages” (Table 2). The change package consists of which change ideas will be tested in what way and a prediction of what will happen.

**Do:** In each country, the knowledge platforms organize local learning sessions with a broader stakeholder group to validate and implement the change strategies during each action period. During the action period, the knowledge platforms and the local participants gather data and document challenges and opportunities.

**Study:** By creating a story board, the knowledge platforms study their results, compare these to their predictions, and summarize their learnings. The knowledge platforms present the story boards at the end of each action period during the following international and local learning sessions (Supplement 2).

**Act:** During the next international learning session, the knowledge platforms share reflections; learn from each other; and discuss whether to adopt, adapt, or abandon change ideas. This process is repeated by the knowledge platforms with their local stakeholders during the national learning session. Thereafter, the PDSA cycle is finished, and the experiences feed into the next PDSA cycle.

For CRIM-KT, the collaborative approach concept was applied for the first time across different country contexts, with a focus on knowledge translation in the field of SRHR. The Figure shows the CRIM-KT approach design. Supplement 1 contains additional details on the preparation and implementation phases.

**FIGURE:**
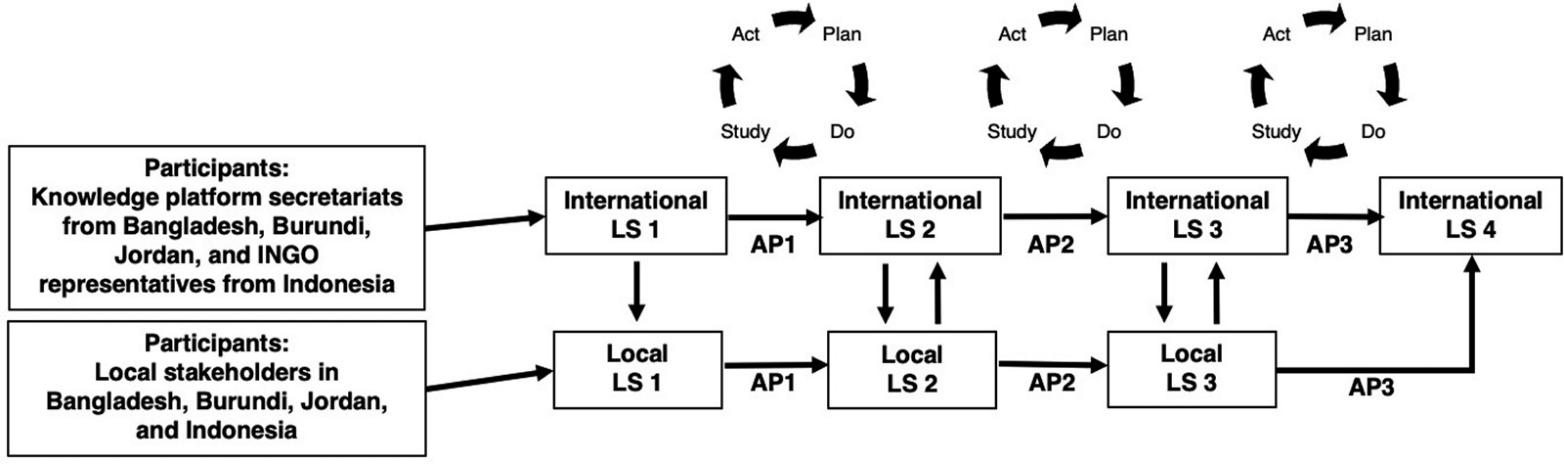
CRIM-KT Applied by SRHR Knowledge Platforms in Bangladesh, Burundi, Indonesia, and Jordan Abbreviations: CRIM-KT, Collaborative Rapid Improvement Model for knowledge translation; AP, action period; LS, learning session; SRHR, sexual and reproductive health and rights.

### Preparation Phase

The preparation phase included writing the CRIM-KT concept note (Supplement 3). Share-Net International appointed a project team to prepare and implement the rapid improvement model. This team comprised a project manager, a chair providing guidance on the CRIM-KT methodology, and 5 knowledge experts in the field of SRHR and knowledge translation from different national and educational backgrounds. Two people from each country knowledge platform secretariat were asked to become knowledge platform representatives and participate and coordinate the design and implementation of the CRIM-KT approach. The knowledge platform representatives set up country teams consisting of staff of their secretariat and local stakeholders.

Share-Net International asked knowledge platform representatives to collaborate with their knowledge platform members and select a topic for SRHR knowledge translation using the following criteria: (1) current policy and practice deviates from best available scientific knowledge; (2) evidence exists describing the gap between research, policy, and practice; (3) examples of better performance exist and are believed to be transfer-able to other settings (Table 1). The knowledge experts advised that all platforms would (preferably) work on a similar or closely related topic to learn from each other's experiences more easily so the secretariats of Bangladesh, the Netherlands, and Jordan chose child marriage and the knowledge platform representatives in Burundi selected teenage pregnancy.

**TABLE 1.** Proposed SRHR Focus Topics in Each Country Knowledge Platform

Share-Net Bangladesh	Share-Net Burundi	Share-Net Jordan	Share-Net Netherlands
Child marriage	Teenage pregnancy	Child marriage	The knowledge platform in the Netherlands did not propose SRHR focus topics but followed the joint decision made by the other knowledge platforms to focus on child marriage or teenage pregnancy. The Dutch knowledge platform identified a member organization willing to implement CRIM-KT with a focus on child marriage in Indonesia.
Comprehensive sexuality education for adolescents	Sexual and gender-based violence	Youth-friendly reproductive health services	
		Reproductive and sexual health education for youth	

Abbreviations: CRIM-KT, Collaborative Rapid Improvement Model for Knowledge Translation; SRHR, sexual and reproductive health and rights.

### Implementation Phase

CRIM-KIT was implemented over 17 months (between September 2017 and January 2019) (Figure). The international learning sessions brought representatives of all country knowledge platforms together to exchange learnings from testing different knowledge translation and use strategies in different contexts. The country knowledge platforms in the Netherlands, Bangladesh, and Jordan alternated in hosting and organizing the international learning sessions. This was not possible for Burundi because of the local security situation. Equal power relations were encouraged by making peer-to-peer learning during the international learning sessions prominent.

The international learning sessions convened country knowledge platforms representatives to exchange learning.

Existing evidence from research and programmatic experience (both explicit and tacit knowledge) on child marriage and teenage pregnancy served as the starting point for the first learning session. During the first learning session, the knowledge platform representatives engaged in defining their problem statement, mind mapping, and identifying the root causes of the problem. The platform representatives experimented with different tools to analyze, organize, and present information including a Flow Chart, Fishbone Diagram, Tree Diagram, Interrelationship Diagram, and Radar Chart.^31^ At the end of the learning session, they developed their first change package (Table 2). The knowledge experts presented global best practices to improve policy and practice to reduce child marriage and teenage pregnancy. Furthermore, different types of knowledge translation strategies and knowledge products to improve policy and/or practice were explained by using examples from a knowledge translation toolkit to bridge the know-do gap.^32^

**TABLE 2.** First Change Package Developed by Share-Net Jordan During Action Period 1

**Problem Statement**	Lack of coordination, sharing, translating, and using knowledge and establishment of consensus around possible solutions between different stakeholders to address child marriage in Jordan.				
**Aim**	To effectively share, translate, and use knowledge among stakeholders where consensus is established around evidence-informed policies and practices related to child marriage in Jordan.				
**Key Drivers** ^a^	**Change Ideas**^b^ **(Concepts)**	**Specific Actions**	**Responsibility**	**Output Measure**	**Outcome Measure**
Collaboration and engagement among stakeholders	Stakeholders are actively engaged in a collective effort to address child marriage	Form a steering committee composed of different stakeholders to define roles and responsibilities around reduction of child marriage (Action period 1)	Country team	Established steering committee	Stakeholders executing their defined and agreed-upon roles and responsibilities
		Organize 2 round tables (Action period 1)	Country team	Organized 2 round tables	Stakeholders established consensus on strategies, initiatives, and activities needed to prevent child marriage
		Conduct 2 workshops for stakeholders to develop a national action plan around the recommendations derived from round tables (Action periods 1 and 2)	Country team	Conducted 2 workshops Developed draft national action plan	Strategies, initiatives, and activities around child marriage are adopted in stakeholders' plans
		Finalize national action plan (Action period 1 and 2)	Country team and local stakeholders	Finalized national action plan around the recommendations that have been approved by the cabinet	Initiatives and/or interventions are implemented jointly by different stakeholders

a Key drivers are the conditions that need to be in place to achieve the goal.

b A change idea is a general notion or approach to change found to be useful in developing specific actions that lead to improvement.

In the second and third learning sessions, the capacity strengthening activities for country platform representatives continued through the delivery of various lectures and exercises by the knowledge experts and the platform representatives themselves. The representatives from Bangladesh facilitated a lecture and exercise on pitching ideas and shared 7 tips for storytelling. The representatives from Jordan shared their experience in policy influencing and writing policy briefs, while the representatives from Burundi shared their experiences performing a literature review. Group exercises using FIRO Behavioral Style Inventory,^33^ the Johari Window Communication Model,^34^ and the Tomas Kilmann Conflict Grid^35^ improved interpersonal relationships, built trust, and increased mutual respect among the participants.

The knowledge experts presented the latest SRHR evidence and provided guidance on how to design and facilitate local learning sessions by adapting content from the international learning sessions to their local context. The knowledge platform representatives performed a stakeholder mapping and analysis and were introduced to different learning styles through the Visual Auditory, and Kinesthetic (VAK) Learning Styles^36^ and Kolb's Experiential Learning Theory to enhance stakeholder collaboration and engagement as well as choose appropriate knowledge translation strategies and develop knowledge products.^37^^,^^38^ Evidence-use mechanisms were discussed to facilitate the uptake of knowledge.^39^ The country knowledge platforms defined change packages that described knowledge translation strategies and envisioned changes in policy and practice to be implemented in the following action period. Key components of these learning sessions were when country platforms shared change ideas that worked and did not work and experimentation with applying ideation techniques like Brainwriting, Six Thinking Hats, Wishing, Opportunity Redefinition, and Semantic Intuition to generate, develop and communicate new ideas to facilitate change.^40^^,^^41^

During the fourth international learning session, country knowledge platforms reflected on the results of this approach in terms of changes in policy and practice and discussed the sustainability of the strategies implemented. In between the international learning sessions, in-country action periods took place in which the country knowledge platforms collaborated with local stakeholders, such as policy makers, researchers, and nongovernmental organization (NGO) representatives, for whom the local secretariat staff organized local learning sessions. Country-level stakeholders reviewed, further developed, and implemented the change packages with support from the country platforms to ensure ownership of the results and sustainability.

Supplement 4 includes the international learning session agendas.

### Evaluation Process

Throughout the CRIM-KT implementation, the knowledge platform representatives and knowledge experts used a participatory action learning process to ensure that learnings about the process could provide inputs for a possible next action learning cycle. During the implementation of CRIM-KT, the knowledge experts conducted a desk review and performed an analysis of the results of each international and local learning session and the action periods based on project documentation (such as storyboards, change packages, and reports). Furthermore, the facilitation team observed the experiences and learning among the country participants and made adaptations for the next action learning period. For example, after the first PDSA cycle, knowledge experts focused more on the adaptation of the content of the international learning sessions to be used in the local learning sessions and the facilitation skills of knowledge platform representatives.

An external researcher (RB) from Erasmus University who was not involved in the implementation of CRIM-KT was invited to participate in the end evaluation. A desk review was done for all project documentation. In-depth interviews were conducted with representatives of the country platforms involved in the CRIM-KT (n=14), and 1 focus group discussion (FGD) was conducted with the knowledge experts (n=4) and the CRIM-KT project coordinator (n=1). The interviews and FGD covered the following topics: (1) implementation: description of activities at international and country level; (2) outcomes: changes in policy, practice, and knowledge translation capacity among country hubs; (3) explanatory factors: characteristics of CRIM-KT method itself and context factors such as sociopolitical situation, gender issues, and power among stakeholders; and (4) crosscutting topics: challenges, lessons learned, and recommendations for improvement.

The interviews lasted about 1 hour and the FGD lasted 2.5 hours. Interviews were recorded and transcribed accordingly. In line with the interview topics, a coding framework was developed in Microsoft Excel to code all data. Researchers from KIT Royal Tropical Institute performed the data analysis and did the triangulation by combining the findings from the desk review, interviews, and FGDs to conduct an overall analysis to draw the main findings.

### Ethics Approval

An ethical waiver was received from the KIT Royal Tropical Institute Ethical Commission. All participants gave informed consent before participating in the interviews and focus group discussion.

## ACHIEVEMENTS OF THE APPROACH

### Capacity Strengthening and Collaborative Action Learning Among Knowledge Platforms

CRIM-KT increased the capacity for knowledge sharing and translation, as well as cross-learning among all country knowledge platforms. The platform representatives mentioned they learned about using PDSA cycles to test change, using tools to analyze root causes and present information to stakeholders, applying knowledge translation techniques in different contexts (including the development and exchange of new knowledge products between country knowledge platforms), and facilitating local learning sessions.

All knowledge platform coordinators indicated that they had learned from the process by studying challenges and subsequently developing new ideas using various ideation techniques.

*We learned how you can identify, in a collaborative way, the root cause of a certain problem and then build up ideas on that. And how you can present your data using different charts.* —Bangladesh knowledge platform coordinator

*In CRIM-KT, we learned different models to conduct a very deep analysis of any key issues that we would like to understand well.* —Burundi knowledge platform coordinator

All knowledge platform coordinators indicated that they had learned from the process by studying challenges and subsequently developing new ideas using various ideation techniques.

Key learning points for the knowledge platform coordinators included the improved ability to test new ideas about knowledge sharing and translation approaches and the development of skills on how best to approach and convene stakeholders.

*We used many tools that we learned a lot from: the different ways of sharing ideas, tools to generate ideas, how to make a community of practice, also how to build partnerships between our stakeholders.* —Jordan knowledge platform coordinator

The process allowed platform coordinators to learn about their own capacities including personality types and learning styles through the international learning session exercises.

*The whole process, the whole CRIM-KT was very nice. It was sort of building of people… The idea was that first you reflect on yourself to become a better facilitator… That helps in how you address your local stakeholders. It also contributed to the other objective that we wanted the country platforms to work more together on and get to know each other better. By doing all those types of exercises, they really got to know each other's personality; they were more free and open. And it helped them, because I see them connected better and they became family. They now ask each other for advice.* —Knowledge expert from the Netherlands

In general, the learning was perceived to be both active and passive.

*CRIM-KT is not only just passive learning. It's also going there, implementing, and following up. That was very good. And then we came back and shared what we have done, what worked, and what did not. This was also very good for my team.* —Bangladesh coordinator

Coordinators of the different platforms were able to inspire each other by modeling effective tactics and approaches. For example, after the Jordan coordinator noticed that Bangladesh was able to involve the private sector, they made similar efforts to involve the private sector in their local context. Additionally, participants in the international collaboration saw that local learning session participants acquired greater levels of understanding of knowledge translation and use and subsequently placed a higher value on the need for knowledge management.

### Changes in Policy and Practice for SRHR

Table 3 shows the change strategies implemented during the 3 learning cycles and the related achievements in SRHR policy and practice that occurred over project implementation in each country. Each country focused on its own areas of attention within their knowledge-sharing and translation activities. Country knowledge platform case studies can be found in Supplement 5.

**TABLE 3.** CRIM-KT Outcomes at Country Knowledge Platform Level, Including Knowledge Products and Knowledge-Sharing and Translation Strategies

Share-Net Bangladesh	Share-Net Burundi	Share-Net Jordan	Share-Net Netherlands in Indonesia
Created an information hub for organizations working on child marriage on the knowledge platform's website. Shared list of organizations and their best practices to prevent child marriage in Bangladesh.	Conducted a mapping and analysis of stakeholders working on the issue of adolescent pregnancy in Burundi.	Developed a national action plan to end child marriage to provide a general framework for limiting the marriage of individuals under 18. This included a 5-year strategy for countrywide interventions.^46^ Presented the action plan successfully to the Prime Minister, endorsed by the Cabinet, and shared with the relevant ministries to allow them to incorporate the proposed interventions in their annual plan if their budget allowed.	In collaboration with the district working group, organized multiple meetings with stakeholders, including traditional and religious leaders, government authorities, parents, and young people. Developed and launched a guideline on the local merarik cultural practice.^50^ Through advocacy efforts, included this guideline as an attachment in the Local Regulation of Child Marriage Prevention that had just been issued by the Parliament.
A booklet titled *Highlights of an initiative addressing child marriage in Bangladesh* was published and disseminated.^42^	Organized a joint broadcasting initiative in the form of a talk show on 12 radio stations to raise awareness among the general public of the risks associated with adolescent pregnancy. This activity was financially supported by an external funder.	Held meetings with potential donors to share the 2018–2022 national action plan and to explore funding opportunities for the different interventions included in the plan.	Developed a syllabus for training on the merarik guideline. Facilitated training for 40 village heads. Integrated training of the village heads on the merarik guideline into the YES I DO program.
A roundtable discussion with policy makers, researchers, and practitioners was held to share the lessons learned in promoting the issue of child marriage in Bangladesh was organized.	Performed a desk review: *Known best interventions to reduce or prevent adolescent pregnancy in Burundi, Kenya, Ethiopia, and the Demographic Republic of Congo*, and a position paper: *How the joint program should or might improve adolescent pregnancy in Burundi.*	With external funds, held a workshop on the mechanisms for integrating child marriage into the plans of civil society institutions and societies.	The Department of Women's Empowerment and Family Planning of West Lombok allocated a budget to expand the coverage of training in the villages outside the 5-year YES I DO program.
The country platform organized a knowledge fair on SRHR where stakeholders shared their knowledge products and efforts to address child marriage and other SRHR issues in Bangladesh.	Held a round table discussion and published a report: *Analyzing the current mechanisms of monitoring adolescent pregnancies in Burundi*,^43^ in collaboration with the National Reproductive Health Program and the National Health Information Management System.	A multistakeholder partnership raised their own funds and focused on awareness training in hospitals, research on the economic and social drivers of child marriage outreach to Syrian schoolgirls, and an awareness campaign at 60 schools.	Added the merarik guideline as an attachment to the Local Regulation of Child Marriage Prevention issued by the parliament in 2020.

Abbreviations: CRIM-KT, Collaborative Rapid Improvement Model for Knowledge Translation; SRHR, sexual and reproductive health and rights.

#### Bangladesh

During the first local learning session with stakeholders in Bangladesh, the knowledge platform identified a lack of collaboration between civil society organizations and policy makers and the absence of knowledge on the consequences of child marriage (both physical and mental) due to a lack of concerted effort. These knowledge and collaboration gaps resulted in limited knowledge sharing and translation into effective interventions to address child marriage. The knowledge platform secretariat and its members selected this issue as the Bangladesh platform's main topic, based on the outcomes of a mind-mapping exercise.

**Figure:**
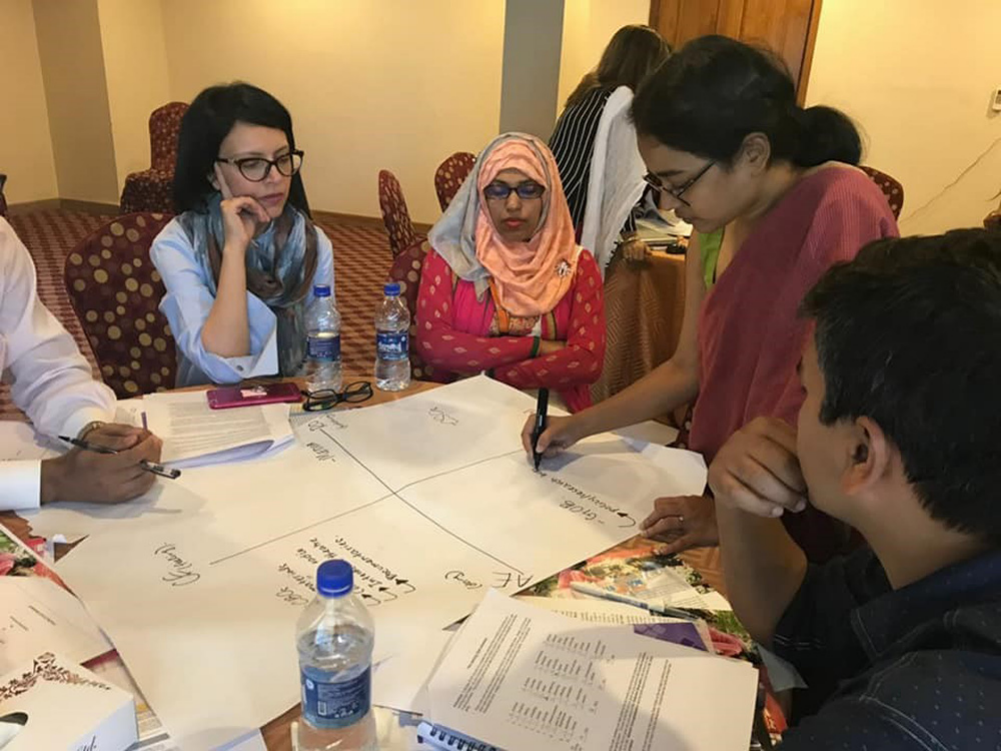
Participants in Bangladesh work on a stakeholder mapping during a local learning session. © 2018 Share-Net Bangladesh

During the second local learning session, the knowledge platform presented the Kolb learning style theory. The participating stakeholders used the learning styles to design knowledge translation activities. The knowledge platform and local stakeholders decided to create an information hub on the knowledge platform website to provide an overview of all organizations working on child marriage in Bangladesh. The knowledge platform and local stakeholders used the results of the stakeholder mapping and analysis to identify these organizations. A booklet highlighting initiatives addressing child marriage in Bangladesh was published and disseminated.^42^ During a roundtable discussion organized by the knowledge platform representatives, the CRIM-KT methodology helped facilitate the identification of knowledge gaps and their translation into explicit knowledge. The local stakeholders were impressed by the methodology and expressed their interest in participating in such collaboration to address different SRHR issues.

CRIM-KT methodology helped facilitate the identification of knowledge gaps and their translation into explicit knowledge.

After CRIM-KT was implemented in Bangladesh, practices changed including strengthened coordination and stimulation of collective impact and collaboration among policy makers, researchers, and practitioners from NGOs and civil society organizations to prevent child marriage. CRIM-KT has helped to create a culture of knowledge sharing. Participants of the learning sessions explicitly mentioned that they were taught to keep the knowledge within the organization because this is the capital of the organization. Throughout the sessions, they became increasingly aware of the importance of knowledge sharing among civil society organizations. After the completion of the CRIM-KT trajectory, the participants joined the existing community of practice on child marriage to continue collaboration and exchange. The collaboration between the community of practice on child marriage, which is facilitated by the Bangladeshi knowledge platform, and government officials has intensified and facilitated evidence-informed working. As a result, the government-level policy makers frequently participate in meetings with civil society organizations and universities to discuss and exchange knowledge related to child marriage. This is a tangible achievement of the CRIM-KT process in Bangladesh.

**Figure:**
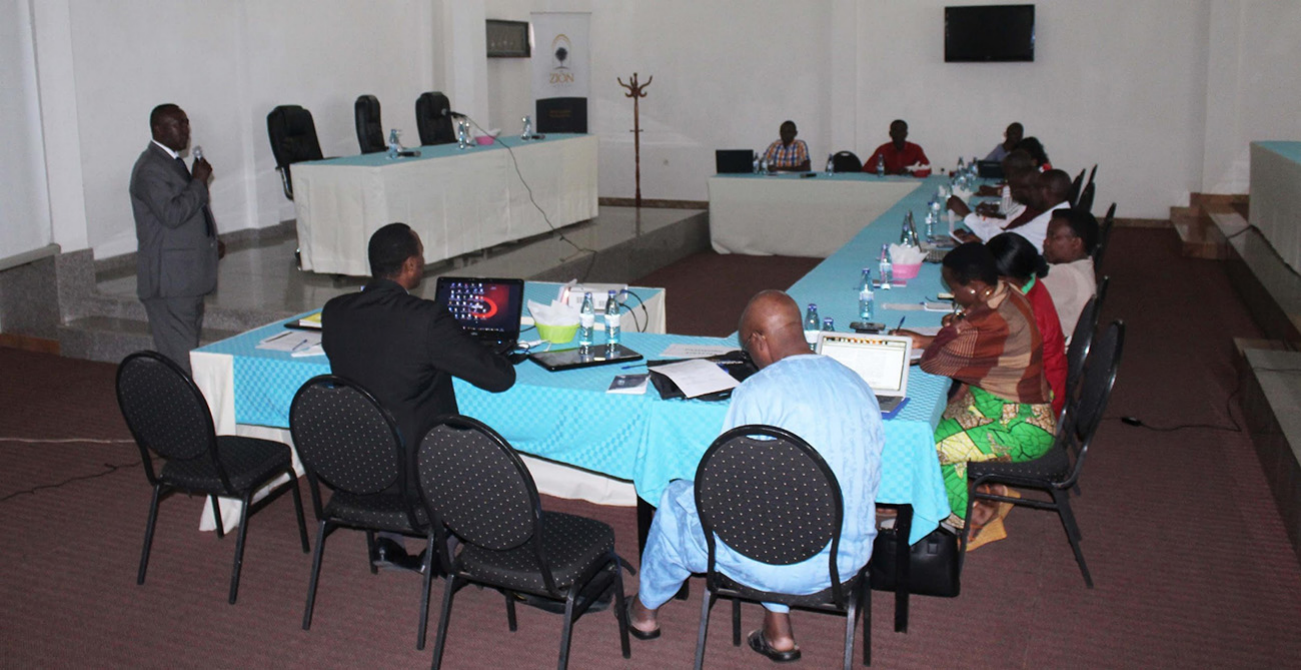
Participants in Burundi learn about the CRIM-KT theory during local learning session 2. © 2018 Share-Net Burundi

#### Burundi

In collaboration with the Reproductive Health Program of Burundi (PNSR), the knowledge platform representatives in Burundi organized 3 local learning sessions that raised awareness among policy makers and other stakeholders of the lack of attention devoted to teenage pregnancy. The learning sessions provided a space for stakeholders to exchange experiences and intentions and organize joint activities. In Burundi, scant evidence was available on the topic of teenage pregnancy.

Through a mind-mapping exercise, the knowledge platform representatives, the PNSR, and the National Institute of Public Health identified knowledge generation as a key priority. As a starting point for changing policy and practice, the knowledge platform representatives with technical support from Share-Net International conducted a mapping and analysis of stakeholders working on teenage pregnancy. Based upon the developed problem statement and a needs assessment, the knowledge platform initiated and coordinated a desk review of international literature on teenage pregnancy interventions, which an intern at Share-Net International performed. In the third action period, knowledge platform representatives held a separate roundtable discussion that resulted in a report written in collaboration with the PNSR and the National Health Information Management System.^43^ The report included recommendations on how the existing government health information system can improve the documentation of adolescent pregnancy at the national level.

CRIM-KT fostered collaboration between stakeholders within a short time frame with clear outputs in the form of different knowledge products. No tangible or measurable changes in policy and practice were observed in Burundi. However, during the second learning session, participants engaged in a discussion with government representatives on a recently introduced policy that banned pregnant girls from attending school.^44^ During the session, Ministry of Education representatives promised to brief the Minister on the participants' recommendation to repeal the ban. A week after the learning session, the Burundian Ministry of Education reversed this ban.^44^ The country knowledge platform team assumes that the discussion during the second learning session partly contributed to this policy reversal.

CRIM-KT fostered collaboration between stakeholders within a short time frame with clear outputs in the form of different knowledge products.

#### Jordan

In Jordan, the knowledge platform's production of a detailed study and policy brief on child marriage before the implementation of CRIM-KT supported evidence-informed decision making.^45^ This evidence led to the approval of the national action plan by the Prime Minister. The knowledge platform together with its members selected child marriage as its topic because evidence on its harmful consequences already existed. Based on a mind-mapping exercise during the first local learning session, the knowledge platform representatives and participating stakeholders prioritized that more collective efforts were needed to ensure that different actors, including the government, took collaborative action to implement policies and programs to address child marriage. In the second learning session, the knowledge platform presented the VAK learning styles and Kolb Experiential Learning Style theory, and participants mapped stakeholders according to their learning styles.

**Figure:**
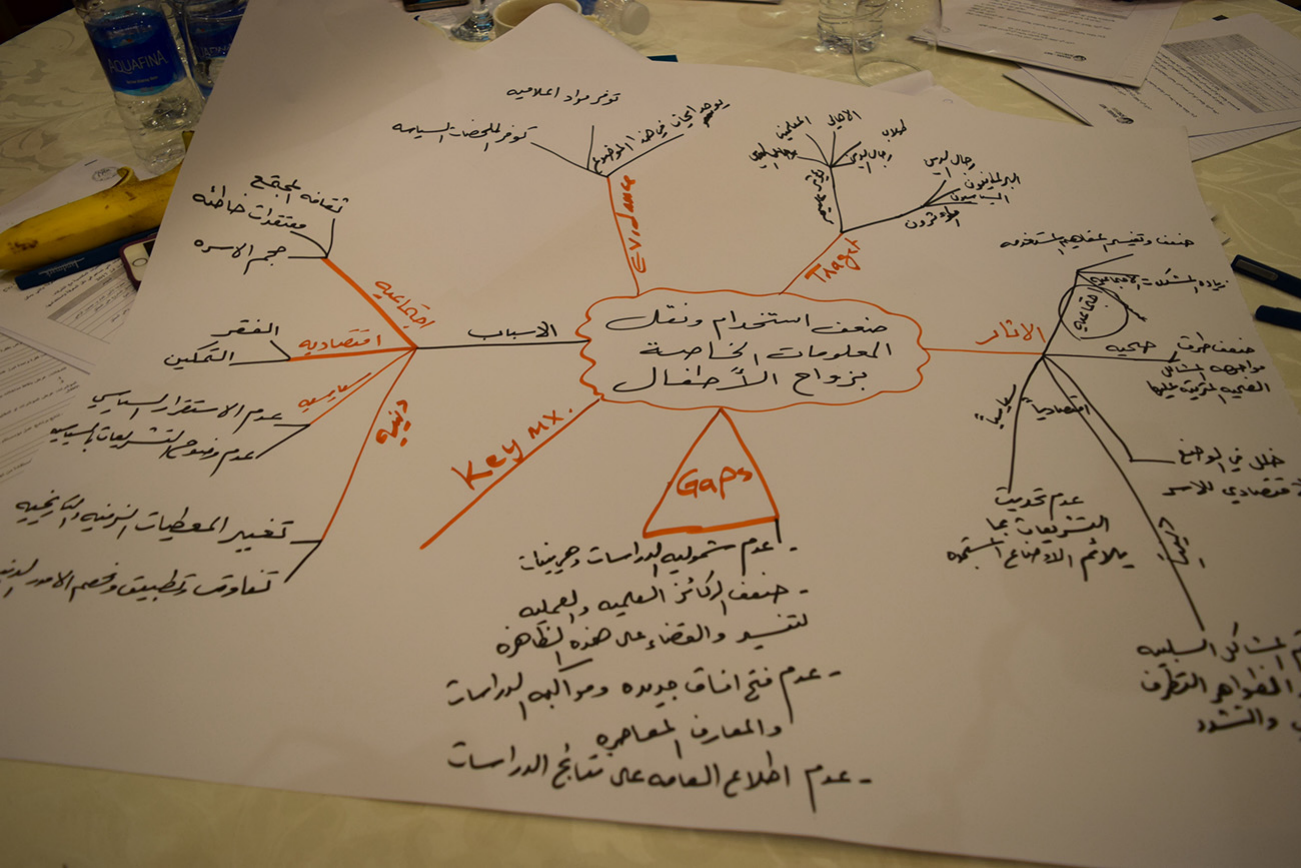
Participants in Jordan created a mind map of child marriage during local learning session 1. © 2017 Share-Net Jordan

Due to the implementation of CRIM-KT, stakeholders, including governmental and NGOs, participated in the local learning sessions. The stakeholders conducted a root-cause analysis of the increased child marriage in Jordan that highlighted the multifactorial causes that need national-level efforts to address it, and accordingly, they developed a national action plan^46^ that outlines policies and programs to prevent child marriage. The outcomes of the VAK and Kolb learning exercises were used to decide stakeholders' roles and contributions in the national action plan. The Secretary-General of the Higher Population Council, where the Jordan knowledge platform is hosted, presented the national action plan to the Prime Minister. After receiving the Prime Minister's approval, the Cabinet endorsed the action plan, including the recommendation to increase the legal age for marriage from age 15 to 16 years.^46^ As mandated by the government, the Higher Population Council handed over responsibility for following up on the national action plan implementation to the National Council for Family Affairs.

The limited funding available from governmental and NGOs to implement the activities recommended by the action plan was a challenge. To address this, the knowledge platform held several meetings with potential donors to explore funding opportunities for the different activities outlined in the plan. With external funds, the knowledge platform representatives conducted a workshop on the mechanisms for integrating child marriage prevention into the action plans of civil society institutions and societies. Changes in practice took place after the King Hussein Foundation formed a multistakeholder partnership, which independently raised funds and implemented joint activities.

Knowledge platform representatives conducted a workshop on the mechanisms for integrating child marriage prevention into the action plans of civil society institutions and societies.

#### Indonesia

In Indonesia, an INGO, as part of the 5-year YES I DO Child Marriage Alliance program in West Lombok, implemented CRIM-KT.^24^ The INGO representatives identified a theme that addressed a local interpretation of the *merarik* (or *merariq*)—meaning marriage in Sasak—a unique cultural tradition (i.e., elopement) of the Sasak ethnic community in West Lombok that supports the “kidnapping” of young girls. This practice contributes to a high incidence of child marriage and a related high divorce rate.^47^^,^^48^ Traditionally, the woman's parents ask for their daughter to be “abducted/kidnapped.” By using the word “abduct,” parents convey the message that their daughter should be guarded and protected by the man their daughter truly loves. After a man and woman meet, the man visits the woman's house to obtain her consent and approval to initiate the merarik.^49^ The traditional form of the merarik cultural practice does not encourage child marriage.^48^ However, nowadays, the practice is used to facilitate underage marriages and is often done without the permission of the girl and/or her parents. Regular laws do not apply, and an underage person can legally get married with their village head's permission.

**Figure:**
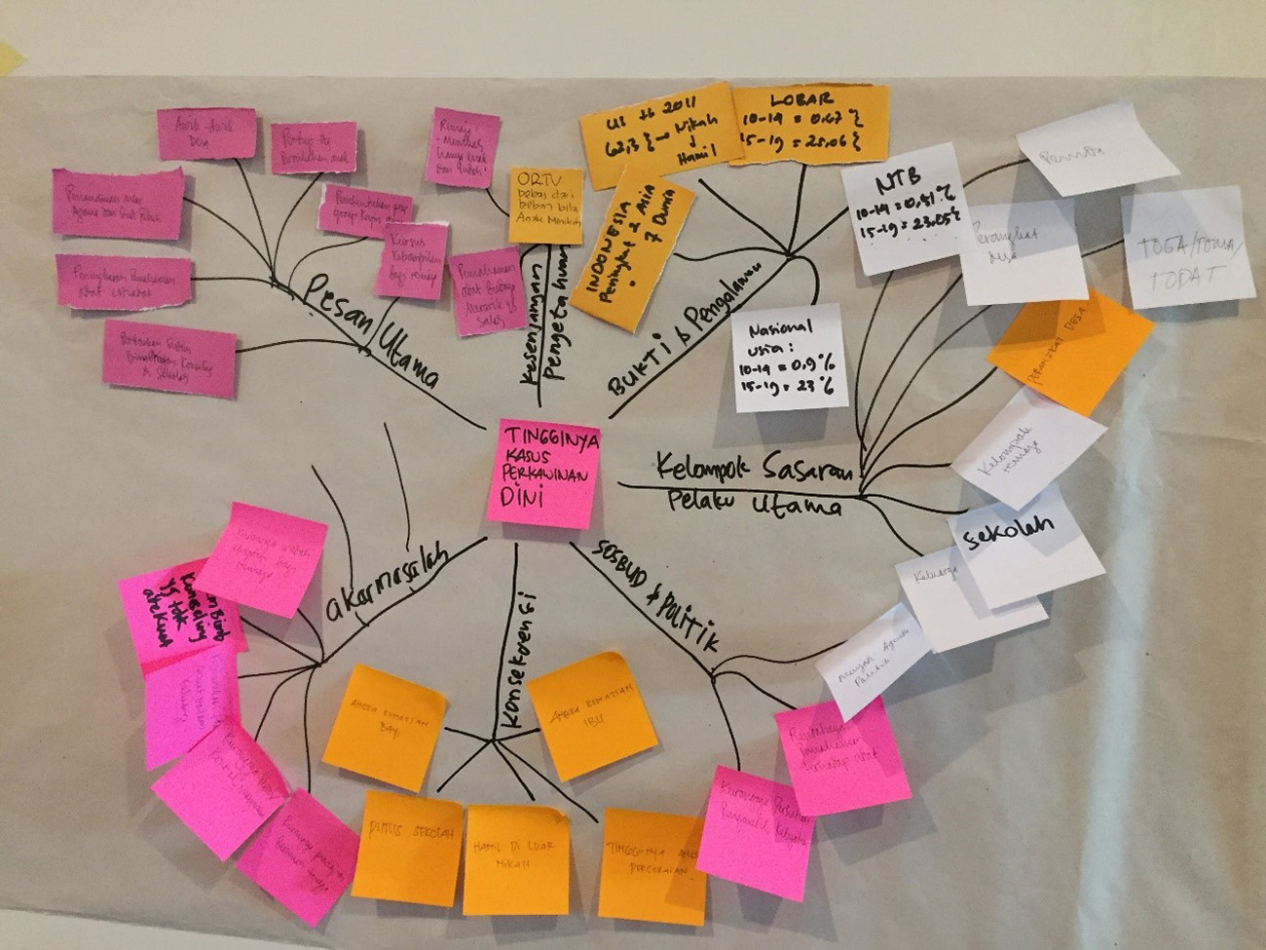
During a break-out exercise in learning session 1, participants in West Lombok, Indonesia, created a mind map to present information on child marriage. © 2018 Rutgers Indonesia

The implementation of CRIM-KT contributed to reinforcing both available knowledge on the harmful consequences of child marriage and alignment with local government priorities to prevent child marriage. By building on the interests of the existing multistakeholder district working group, the implementing INGO and another member organization of the YES I DO Alliance organized a series of meetings with stakeholders including traditional and religious leaders, government authorities (District Health Office, District Education Office, District Office of Population Control, Family Planning, Women's Empowerment, and Child Protection), academicians, young people, and parents. During these meetings, stakeholders agreed that the harmful interpretation of the merarik cultural practice was a root cause of the high incidence of child marriage in Lombok. To identify this root cause, stakeholders performed a force field analysis and fishbone root cause analysis and created a radar chart and tree diagram during the first local learning session. The leading INGO and district working group members developed a technical guideline to clarify that the traditional concept and process of merarik does not encourage underage marriage.^50^

Stakeholders used the VAK learning styles and Kolb experiential learning style theory to identify their own learning style and the learning style of the village heads and traditional leaders. They used the outcomes to choose the most appropriate methods for training on the merarik guidelines. Subsequently, traditional leaders trained village heads in Lombok to support them in refusing to grant permission for child marriages. The trainings were supported by the District Office of Population Control, Family Planning, Women's Empowerment and Child Protection and the INGO. The INGO integrated the training of village heads on the guideline into the 5-year YES I DO program. In 2019, the local guideline on merarik was integrated into the existing Local Regulation of Child Marriage Prevention issued by the parliament in West Lombok. This can be characterized as a long-term sustainable change.

## LESSONS LEARNED: FACTORS CONTRIBUTING TO CHANGES IN POLICY AND PRACTICE

Based on the interviews with knowledge platform participants and knowledge experts, changes in policy and practice can be attributed to the CRIM-KT methodology itself, as well as by the developments in the country context (Table 4).

**TABLE 4.** Factors Contributing to Changes in Policy and Practice, Related to CRIM-KT and Country Context in Bangladesh, Burundi, Indonesia, and Jordan

	Related to CRIM-KT	Related to country context
Enabling factors	Participatory approach involving stakeholders Systematic and structured approach, allowing for contextual adaptation Short time frame, creating a sense of urgency Efficient tools such as simple change package format that forced prioritization of objectives Openness about making mistakes or admitting that strategies do not work Time for reflection through storyboards Cross-learning on 2 levels (international and country collaboration teams) New methods for stakeholder involvement Transparency of international and local activities and involvement of coordinators in program Feelings of pride in sharing successes with other platforms during international learning sessions	Personal connections between stakeholders Strong knowledge platform secretariat Embedding/structure of knowledge platform: e.g., being a government institution working directly for Prime Minister (Jordan) Availability of existing multistakeholder platforms Embedding in other projects (e.g., YES I DO program) and leadership of local government staff (Indonesia) Ability to talk to government authorities Alignment with government priorities (Jordan and Indonesia) Relatively low costs for organizing meetings (Indonesia) Alignment with local priorities (Indonesia)
Disabling factors	Short time frame Limited capacity strengthening for facilitation of local collaboration Limited funds available to develop and organize activities during the local action periods Content of some international learning sessions less applicable to local (community) level Geographical distance between knowledge platform and international knowledge experts providing communication/support Limited funds available to develop and organize activities during the local action periods	Natural disasters (earthquake in Lombok) Staff turnover among stakeholders and within knowledge platform participants in CRIM-KT (Indonesia and Jordan) Power dynamics among local stakeholders Staff of knowledge platform falling ill (Bangladesh and Burundi) Lack of government willingness to collaborate with nongovernmental organizations (Burundi) Resistance of religious actors to address child marriage (Indonesia and Jordan) Limited capacity of knowledge platform to produce research (Burundi) Limited funding available from government and/or other stakeholders to financially support the implementation of activities proposed in local learning sessions (Burundi and Bangladesh) Political context: e.g., elections and the office was closed in Burundi for some time Language barriers: English level of Burundi staff hampered full participation in international learning sessions held in English

Abbreviation: CRIM-KT, Collaborative Rapid Improvement Model for Knowledge Translation.

Key enabling factors linked to the CRIM-KT methodology are the combination of flexibility and structure and the limited time frame (which worked as a “pressure cooker”). Contextual factors that contributed to outcomes included the capacity of the institutions leading the change process, alignment with local policy priorities, close collaboration with government entities, and embedding in (existing) multistakeholder partnerships.

Fragile sociopolitical contexts and a limited evidence base, as seen in Burundi, were found to hamper the contribution of the CRIM-KT methodology to changes in SRHR-related policy and practice.

## COMPARISON OF FINDINGS WITH OTHER KNOWLEDGE TRANSLATION APPROACHES

Compared to other knowledge translation approaches,^51^ CRIM-KIT seems more flexible and adaptable to the needs of the specific contexts in which it is used by developing tailored strategies to reach the target audience. Fafard and Hoffman acknowledge the importance of tailoring approaches to the needs of audiences, the policy context, and policy instruments.^52^ Also, Liverani et al.^53^ recognize the importance of institutional characteristics and political considerations. The Overseas Development Institute^54^ reckons in its guidance that policy development is not a linear process and that methods should, therefore, allow for flexibility.

CRIM-KT uses research and programmatic evidence and considers local priorities relevant to the country's policy context as the starting point. CRIM-KT differs from other knowledge translation approaches in its focus on experiential exchange across different countries. The swift and structured approach has likely led to the acceleration of learning by experimenting with knowledge translation strategies and sharing experiences and lessons learned in a cyclical fashion. This can lead to quick improvements over relatively short time periods.

CRIM-KT uses research and programmatic evidence and considers local priorities relevant to the country's policy context as the starting point.

Knowledge translation practitioners currently use an abundance of rapid knowledge translation models.^4^^,^^14^^,^^39^^,^^55^^,^^56^ For example, in the Integrated Knowledge Translation Approach, researchers and research users have to be involved in all parts of the research process.^55^ In rapid review services, researchers provide these services to inform emergent decisions faced by decision makers in health care settings.^56^ Helpdesks are an approach in which a research institution provides rapid evidence-on-demand advice to policy makers. These helpdesks can answer specific questions, provide a brief literature review, or include summaries or comments from subject experts.^39^ These rapid models have a more linear approach to moving from evidence to action. They also have a passive nature and do not allow for frequent refocus on knowledge translation questions.^15^ In contrast, through the cyclic nature of the learning cycles, CRIM-KT ensures the option to adapt the knowledge questions based on stakeholders' input and learnings from other countries.

Furthermore, most of these rapid models do not address the institutionalization of knowledge translation work. CRIM-KT focuses on achieving more lasting change by working with existing knowledge platforms and their stakeholders to institutionalize evidence-informed decision making in policy, practice through focusing on the exchange of knowledge among platforms, and incorporating capacity strengthening on the use of different knowledge translation strategies into the learning sessions and action periods. In addition to research, crucial elements in CRIM-KT include technical advice from experts and civil society participation to inform deliberative policy dialogues, which are often overlooked forms by rapid knowledge translation methods for policy making in health systems.^57^

Malla et al.^51^ mention the importance of considering the global-local dynamics, links between power and knowledge, and contextual factors that influence capabilities in low- and middle-income countries to access, generate, and utilize evidence. CRIM-KT partly addressed these issues by organizing learning sessions at both the international level (across countries) and local level (within countries). Allowing the country knowledge platforms to alternate hosting and organizing the international learning sessions enabled peer-to-peer learning, encouraged equal power relations, allowed global and local evidence to be incorporated, and country-level stakeholder perspectives and experiences to be included. Local stakeholder engagement was achieved through stakeholder mapping and analysis. By using CRIM-KT, the knowledge platforms created a dynamic context in which stakeholders from different backgrounds had access to knowledge and a voice to advocate for change.

CRIM-KT facilitated demand-driven and locally led knowledge translation strategies by the country knowledge platforms, which strengthened local capacities and institutions.^58^ The country knowledge platforms and their stakeholders defined knowledge translation priorities that were not imposed at the global level. Secondly, the country platforms facilitated their own local learning sessions, designed their own change packages, and implemented their own knowledge translation strategies together with their stakeholders.

CRIM-KT facilitated demand-driven and locally led knowledge translation strategies by the country knowledge platforms, which strengthened local capacities and institutions.

A power-interest matrix was used to map power imbalances between stakeholders. Group exercises, using FIRO Behavioral Style Inventory, the Johari Window Communication Model, and the Tomas Kilmann Conflict Grid, addressed these power imbalances by improving the interpersonal relationships of stakeholders by building trust and mutual respect. The international learning sessions were designed by the knowledge experts in collaboration with and based on the needs of the knowledge platform representatives with space for the country platforms to share their expertise and knowledge regarding knowledge translation strategies, which facilitated cross-country peer-to-peer exchange. In this way, CRIM-KT provided opportunities for 2-way learning and exchange, where both the international and local country collaboration teams learned and benefited, challenging the “paradigm of uni-directional problem solving.”^55^ The collaboration and team building across the countries also increased commitment to creating lasting change.

## RECOMMENDATIONS FOR FUTURE APPLICATIONS

We have identified the following recommendations for improvement.

The available funding for all countries was the same. Funding should be adapted to the costs required in relation to the specific local context and the activities of the change packages.

Connection to and involvement of research institutions within the countries should be strengthened to ensure that the latest evidence is included or generated relatively quickly during the implementation of the project.

A more in-depth gender and power analysis should be included as a tool to better understand how stakeholders act and could be involved to create change in policy and practice.

For future application, a cost-benefit analysis should be performed. The international learning session consumed a relatively large part of the budget, and it should be investigated whether some meetings could take place remotely to save costs. However, the knowledge experts involved indicated that physical meetings should be prioritized over holding all meetings online. Modern technology provides useful options for holding virtual meetings, but the value of face-to-face interaction should not be underestimated since it helps to foster relationship-building, trust, communication, and exchange of lessons learned.

In terms of sustainability, the use of CRIM-KT has contributed to changes in policy and practice, as well as capacity strengthening. More research is needed to assess the implementation of policies, the extent to which they are adhered to, and whether early changes in practice will eventually lead to reductions in the prevalence of child marriage and teenage pregnancy. The existing country knowledge platforms of Share-Net International in Bangladesh, Burundi, Jordan, and the Netherlands have continued their work. In 2020, Share-Net International has expanded and set up additional knowledge platforms in Burkina Faso, Ethiopia, and Colombia. The next round of CRIM-KT started in February 2022 and will focus on improving SRHR information and education.

## CONCLUSION

The Share-Net International Rapid Improvement Model, or CRIM-KT, yielded several changes in SRHR policies and practices across different countries and strengthened the capacity of the knowledge platforms. The key factors that contributed to lasting change were the systematic, structured, and participatory approach, which allowed for contextual adaptation and stakeholder involvement, and the cross-learning on 2 levels (international and country collaboration teams). There is a need to further develop and apply the model beyond the SRHR field to support more rapid knowledge translation in global health.

## Supplementary Material

GHSP-D-21-00461-Supplement3.pdf

GHSP-D-21-00461-Supplement2.pdf

GHSP-D-21-00461-Supplement1-4-5.pdf

## Translation

**RAPPORT D'ACTION SUR LE TERRAIN**

**Titre:** Utilisation d'une approche de traduction rapide des connaissances pour améliorer la santé et les droits sexuels et reproductifs au Bangladesh, au Burundi, en Indonésie et en Jordanie

**Titre abrégé:** Application rapide des connaissances pour la santé et les droits sexuels et reproductifs

Kimberley Meijers, Anke van der Kwaak, Ibrahim Aqel, Robert Borst, Françoise Jenniskens, Aryanti Radyowijati, Arnob Chakrabarty, Eric Nzeyimana, Ely Sawitri, Noor Tromp

Message d'accroche: Il existe un besoin croissant d'approches visant à soutenir les processus rapides de traduction des connaissances qui peuvent créer des changements dans les politiques et les pratiques et qui puissent s'appliquer à différents contextes nationaux. Le modèle collaboratif d'amélioration rapide de la traduction des connaissances en matière de santé et de droits sexuels et reproductifs (SDSR) mis en œuvre dans 4 pays a permis d'améliorer les pratiques et les politiques SDSR.

**Principales conclusions**

- Le modèle collaboratif d'amélioration rapide de l'application des connaissances (CRIM-KT) répond au besoin croissant dans le domaine de la santé mondiale de méthodes pour soutenir les processus rapides d'application des connaissances qui sont à la fois structurés et flexibles, qui incluent des types de connaissances autres que scientifiques et qui favorisent le dialogue et la collaboration entre chercheurs, décideurs, praticiens et acteurs du secteur privé.
- Les principaux facteurs favorables au changement ont été le caractère systématique, structuré et participatif de l'approche du modèle CRIM-KT, qui a permis l'adaptation au contexte et la participation des intervenants, ainsi que l'apprentissage croisé aux niveaux international et national.
- Les principaux facteurs défavorables ont été le court délai entre les sessions d'apprentissage et les fonds limités disponibles pour développer et organiser des activités pendant les périodes d'action locale.

**Implications clés**

- Ce modèle d'amélioration rapide devrait être développé et testé plus avant en s'attaquant aux facteurs handicapants et en l'appliquant au-delà du domaine des SDSR.
- Les décideurs politiques sont encouragés à stimuler et à participer aux approches d'amélioration rapide menées par les plateformes de connaissances et à utiliser les résultats spécifiques au contexte et fondés sur des preuves dans l'élaboration des politiques SDSR.

**En Français**

**Utilisation d'une Approche de Traduction Rapide des Connaissances pour Améliorer la Santé et les Droits Sexuels et Reproductifs au Bangladesh, au Burundi, en Indonésie et en Jordanie**

**Principales conclusions**

- Le modèle collaboratif d'amélioration rapide de l'application des connaissances (CRIM-KT) répond au besoin croissant dans le domaine de la santé mondiale de méthodes pour soutenir les processus rapides d'application des connaissances qui sont à la fois structurés et flexibles, qui incluent des types de connaissances autres que scientifiques et qui favorisent le dialogue et la collaboration entre chercheurs, décideurs, praticiens et acteurs du secteur privé.
- Les principaux facteurs favorables au changement ont été le caractère systématique, structuré et participatif de l'approche du modèle CRIM-KT, qui a permis l'adaptation au contexte et la participation des intervenants, ainsi que l'apprentissage croisé aux niveaux international et national.
- Les principaux facteurs défavorables ont été le court délai entre les sessions d'apprentissage et les fonds limités disponibles pour développer et organiser des activités pendant les périodes d'action locale.

**Implications clés**

- Ce modèle d'amélioration rapide devrait être développé et testé plus avant en s'attaquant aux facteurs handicapants et en l'appliquant au-delà du domaine des SDSR.
- Les décideurs politiques sont encouragés à stimuler et à participer aux approches d'amélioration rapide menées par les plateformes de connaissances et à utiliser les résultats spécifiques au contexte et fondés sur des preuves dans l'élaboration des politiques SDSR.

**Résumé**

**Introduction:** L'application des connaissances aux politiques et aux pratiques est importante pour prévenir la morbidité et la mortalité liées à la santé et aux droits sexuels et reproductifs (SDSR) et garantir l'accès aux droits. Les approches existantes de la traduction des connaissances sont souvent relativement rigides et supposent implicitement des processus linéaires, ce qui conduit à des processus qui prennent du temps et ne sont pas adaptés aux besoins des pays.

**Approche:** Les plates-formes de connaissances SDSR ont conçu et mis en œuvre un modèle collaboratif d'amélioration rapide de la traduction des connaissances (CRIM-KT) au Burundi, au Bangladesh, en Indonésie et en Jordanie. L'approche consistait en des sessions d'apprentissage et des périodes d'action avec l'objectif d'améliorer les politiques et les pratiques relatives à la prévention du mariage des enfants et des grossesses adolescentes. Pour évaluer l'approche, un processus d'apprentissage par l'action participative a eu lieu tout au long de la mise en œuvre (septembre 2017 et janvier 2019). Une évaluation finale a été réalisée à l'aide d'une étude documentaire du projet, d'entretiens approfondis et d'un groupe de discussion pour documenter le processus, les résultats et les leçons apprises.

**Réussites:** En Indonésie, une politique du gouvernement local a été modifiée et vise à prévenir le mariage des enfants en évitant une mauvaise interprétation de la pratique culturelle locale. En Jordanie, le cabinet a approuvé un plan d'action national pour prévenir le mariage des enfants et des changements de pratique ont eu lieu. Au Burundi, aucun changement tangible n'a été observé dans la politique et la pratique en matière de SDSR. Au Bangladesh, la pratique a changé grâce au renforcement de la coordination en vue d'une collaboration et d'un échange entre les parties prenantes pour prévenir le mariage des enfants. Dans tous les pays, l'approche a considérablement renforcé les capacités de traduction des connaissances des participants.

**Conclusion:** Le CRIM-KT a permis d'améliorer les politiques et les pratiques dans un espace de temps relativement court et dans des contextes différents. Cela peut s'expliquer par l'approche systématique, structurée et participative, permettant l'adaptation au contexte et la participation des parties prenantes, ainsi que par l'apprentissage croisé à 2 niveaux (équipes de collaboration internationale et nationale). Les principes du CRIM-KT peuvent être développés et appliqués dans d'autres domaines de la santé mondiale pour renforcer les processus de traduction des connaissances.

**Peer Reviewed**

**First published online:** April 6, 2022.
