# Supplementary material for: Using a Rapid Knowledge Translation Approach for Better Sexual and Reproductive Health and Rights in Bangladesh, Burundi, Indonesia, and Jordan
Source: Glob Health Sci Pract. 2022 Apr 28;10(2):e2100461. doi: 10.9745/GHSP-D-21-00461 (PMC9053141; doi:10.9745/GHSP-D-21-00461)
Supplement: GHSP-D-21-00461-Supplement3.pdf [file GHSP-D-21-00461-Supplement3.pdf]

Supplement to: Meijers K, van der Kwaak A, Aqel I, et al. Using a rapid knowledge translation approach for better sexual and reproductive health and rights in Bangladesh, Burundi, Indonesia, and Jordan. *Glob Health Sci Pract*. 2022;10(2): e2100461. https://doi.org/10.9745/GHSP-D-21-00461

Share-Net International

SHARE-NET INTERNATIONAL RAPID IMPROVEMENT MODEL (SHIRIM) FOR SRHR KNOWLEDGE TRANSLATION AND USE

Supplement to: Meijers K, van der Kwaak A, Aqel I, et al. Using a rapid knowledge translation approach for better sexual and reproductive health and rights in Bangladesh, Burundi, Indonesia, and Jordan. *Glob Health Sci Pract*. 2022;10(2): e2100461. https://doi.org/10.9745/GHSP-D-21-00461

CONCEPT NOTE FOR STRENGTHENING THE LEARNING CAPACITY OF AND BETWEEN THE COUNTRY HUBS OF SHARE-NET INTERNATIONAL FOR TRANSLATING KNOWLEDGE INTO POLICY AND PRACTICE.

For more information, please contact:

Kimberley Meijers, Share-Net International Country Coordinator & Knowledge Expert

Email: k.meijers@kit.nl

Amsterdam, 2017

Supplement to: Meijers K, van der Kwaak A, Aqel I, et al. Using a rapid knowledge translation approach for better sexual and reproductive health and rights in Bangladesh, Burundi, Indonesia, and Jordan. *Glob Health Sci Pract*. 2022;10(2): e2100461. https://doi.org/10.9745/GHSP-D-21-00461

TABLE OF CONTENTS

ABSTRACT 4

1. INTRODUCTION 5

2. RATIONALE 5

3. PROBLEM STATEMENT & JUSTIFICATION 6

PROBLEM STATEMENT 6
OVERALL AIM 7
OBJECTIVES: 7

4. METHODOLOGY 7

FUNDAMENTAL QUESTIONS FOR IMPROVEMENT: 9
PREPARATION PHASE 10
IMPLEMENTATION PHASE: 11
SUSTAINABILITY PHASE: 12

5. TIMEFRAME 13

Supplement to: Meijers K, van der Kwaak A, Aqel I, et al. Using a rapid knowledge translation approach for better sexual and reproductive health and rights in Bangladesh, Burundi, Indonesia, and Jordan. *Glob Health Sci Pract*. 2022;10(2): e2100461. https://doi.org/10.9745/GHSP-D-21-00461

ABSTRACT

The Share-Net International Rapid Improvement Model (SHIRIM)[1] aims to explore and test strategies for translating knowledge into better SRHR policy and practice in the specific context of each Share-Net country knowledge platforms. This model uses the so-called Collaborative Approach as a structured learning methodology to facilitate collaboration between the country hubs and to stimulate sharing experiences, practices, and lessons learned.

This concept note describes a 15 to 18 month action learning project using SHIRIM. It focuses on strengthening knowledge management and capacity strengthening to support the aims and objectives of Share-Net International and the country knowledge platforms. The outputs of the Share-Net International Rapid Improvement Model will be the knowledge products developed by the Share-Net country knowledge platforms. A minimum of one scientific article will be published about the process of the Share-Net Rapid Improvement Model in a peer reviewed journal.

[1] Originally the name of the designed approach was Share-Net International Rapid Improvement Model (SHIRIM). To expand the reach of this approach the existing name was replaced by a more descriptive name that others can adopt: Collaborative Rapid Improvement Model for Knowledge Translation (CRIM-KT).

Supplement to: Meijers K, van der Kwaak A, Aqel I, et al. Using a rapid knowledge translation approach for better sexual and reproductive health and rights in Bangladesh, Burundi, Indonesia, and Jordan. *Glob Health Sci Pract*. 2022;10(2): e2100461. https://doi.org/10.9745/GHSP-D-21-00461

1. INTRODUCTION

Share-Net International (SNI), the Knowledge Platform on Sexual and Reproductive Health and Rights (SRHR), was established in September 2013. SNI is hosted by KIT Royal Tropical Institute in Amsterdam, the Netherlands. The main goal of SNI is to strengthen linkages between research, policy and practice in the field of SRHR through a) sharing, b) generating, c) translating and d) using knowledge.

The objectives of SNI are to:

1. identify relevant knowledge for achieving improved SRHR;
2. stimulate sharing of existing relevant knowledge among platform participants;
3. generate new knowledge to address prioritized research gaps;
4. translate knowledge into formats appropriate for intended audiences (application of knowledge);
5. understand and facilitate the complex processes by which evidence informs policy, both in the Netherlands and in partner countries,
6. facilitate learning and collaborative exchange among those participating.

Share-Net International encompasses national knowledge platforms in four countries: Bangladesh, Burundi, Jordan and the Netherlands, referred to as 'country hubs'.[2] These national knowledge platforms serve as channels for situation assessments, defining research priorities, facilitating research participation by country institutions and hosting the local knowledge base/platform. They also stimulate the participation of key international partners and regional and national actors in SRHR, based in their countries.

This concept note describes a 15 to 18 month action learning process by building upon the Collaborative Approach implemented in the context of SNI and is called "Share-Net International Rapid Improvement Model (SHIRIM) for SRHR Knowledge Translation and Use." First the rationale will be described, followed by the problem statement and justification, the methodology, approach, outcomes and timeline.

2. RATIONALE

Since SNI was established, the knowledge platform has invested in the development of participatory and inclusive mechanisms in their focus countries, that encourage knowledge management for development within SRHR. The Theory of Change of SNI includes four pathways: knowledge generation, knowledge dissemination, knowledge translation and promotion of knowledge use.

The evaluation of SNI[3] and the review of the five Knowledge Platforms[4] concluded that there was still a need to continue addressing SRHR issues and continue the knowledge brokering processes through exchange, creation and use. The evaluators mentioned in their report that knowledge exchange between the country

---

[2] In 2021 additional Share-Net country hubs were set-up in Burkina Faso, Colombia and Ethiopia
[3] Ewijk v. E, Gent v. M, Michielsen K, Vermandere H. Evaluation of Share-Net International, the knowledge platform on SRHR. Ghent: Kaleidos Research and International Centre for Reproductive Health, Ghent University; 2017.
[4] Lammers E, de Winter D. The Gold Standard: Exploring the added value of the Dutch knowledge platforms, Amsterdam: 2017.

hubs was still limited. The evaluators recommended that SNI should play a more active role in facilitating this exchange and should pay more attention to the pathways of knowledge translation and use. The evaluators advised that the knowledge platforms should work on designing relevant monitoring, evaluation and learning frameworks to strengthen their ability to measure the impact and effectiveness of their knowledge brokering strategies and efforts.

During a country hubs meeting in February 2017, representatives from the four secretariats from Bangladesh, Burundi, Jordan and the Netherlands came together to discuss progress, drawbacks and the way forward. All representatives showed interest to meet more often than once a year and mentioned their specific interest to exchange knowledge and experiences regarding the managing of a knowledge network and their role as knowledge broker, with specific focus on translating knowledge into products for use in changing policy and practice.

Knowledge brokering is the core business of SNI and up until then, the country hubs had focused mainly on knowledge generation and dissemination and to a lesser extent on knowledge translation and use.[5] Examples of knowledge brokering activities that were successfully implemented are the newsletters, websites, thematic meetings, policy briefs, (thematic) working groups[6] and conference debriefs. The country hubs all expressed that they struggled in determining which knowledge translation strategies and knowledge products were most effective in their respective context.

3. PROBLEM STATEMENT & JUSTIFICATION

PROBLEM STATEMENT

Share-Net International and the country hubs have made a lot of progress in developing and implementing knowledge brokering processes with their partners and members in the four countries. Despite the success, that SNI has achieved through the development of participatory and inclusive mechanisms that encourage SRHR knowledge generation, dissemination, translation and promotion of use in the four countries, there was still limited knowledge exchange between the country hubs. The country hubs struggled in determining which strategies and knowledge products were most effective in their respective context.

Share-Net International and its country hubs therefore jointly decided they wanted to invest in developing, implementing, and exchanging additional strategies to translate research findings into knowledge products which successfully move the newly acquired knowledge towards policy influencing and improving practice.

There was scope for **strengthening linkages and collaborations between the country hubs** and SNI. During the Share-Net country hubs meeting in February 2017, all countries hubs indicated being in favour of building upon Collaborative Approach learning system[7] to achieve this.

[5] Lammers E, de Winter D. The Gold Standard: Exploring the added value of the Dutch knowledge platforms, Amsterdam: 2017.
[6] These working groups turned into Communities of Practice in 2018.
[7] The Breakthrough Series: IHI's Collaborative Model for Achieving Breakthrough Improvement. IHI Innovation Series white paper. Boston: Institute for Healthcare Improvement; 2003. (Available on www.IHI.org).

Supplement to: Meijers K, van der Kwaak A, Aqel I, et al. Using a rapid knowledge translation approach for better sexual and reproductive health and rights in Bangladesh, Burundi, Indonesia, and Jordan. *Glob Health Sci Pract.* 2022;10(2): e2100461. https://doi.org/10.9745/GHSP-D-21-00461

OVERALL AIM

SHIRIM **aims** to build upon the Collaborative Approach to translate newly acquired knowledge towards policy influencing and improving practice and to spread this experience across the Share-Net country hubs, with a focus on experimenting with new knowledge translation strategies.

OBJECTIVES:

- Explore different strategies that effectively influence the complex processes by which evidence informs policy
- Improve understanding of which strategies contribute to successful knowledge translation and use in the specific context of each country hub
- Facilitate learning, collaboration and exchange between country hubs
- Develop the capacity of participating staff from the country hubs secretariats with respect to knowledge brokering
- Enhance sharing and applying knowledge about the most effective strategies that contribute to successful knowledge translation and use in the specific context.
- Sustain strategies and approaches that contribute to successful knowledge translation and use

Knowledge here refers to knowledge created through academic or applied research, as well as through the implementation of innovative projects, conducting scoping studies and systematic reviews and tacit knowledge including gathering lessons learned etc. Knowledge brokering refers to *"the iterative process of knowledge creation, exchange and use."* [8]

4. METHODOLOGY

The Collaborative Approach forms part of the Breakthrough Series[9] developed by the *Institute for Healthcare Improvement* that helps organisations close the gap between what we know and what we do by creating a structure in which interested organisations can easily learn from each other and from recognised experts in topic areas where they want to make improvements.

Usually a Breakthrough Series (BTS) Collaborative is a short-term (6 to 18 months) learning methodology that brings together representatives from different organisations to seek improvement in a focused topic area in healthcare.

Share-Net International built on the BTS Collaborative Approach concept (Figure 1) and developed a model called '*Share-Net International Rapid Improvement Model (SHIRIM) for SRHR Knowledge Translation and Use*.'

8 The Gold Standard: Exploring the added value of the Dutch knowledge platforms. Ellen Lammers and Daniëlle de Winter. February 2017.

9 The Breakthrough Series: IHI's Collaborative Model for Achieving Breakthrough Improvement. IHI Innovation Series white paper. Boston: Institute for Healthcare Improvement; 2003. (Available on www.IHI.org).

Supplement to: Meijers K, van der Kwaak A, Aqel I, et al. Using a rapid knowledge translation approach for better sexual and reproductive health and rights in Bangladesh, Burundi, Indonesia, and Jordan. *Glob Health Sci Pract*. 2022;10(2): e2100461. https://doi.org/10.9745/GHSP-D-21-00461

For SNI it was decided that a period of 15 to 18 months is needed to allow the country hubs sufficient time to develop and try out new strategies. Experts on the specific focus area and application experts share their expertise and will assist the country hubs in selecting, testing, and implementing changes.

The learning methodology alternates between learning sessions in which representatives from the country hubs come together to learn about the chosen topic and to plan changes, and action periods in which the teams would return to their own country hub and test those changes in their own setting.

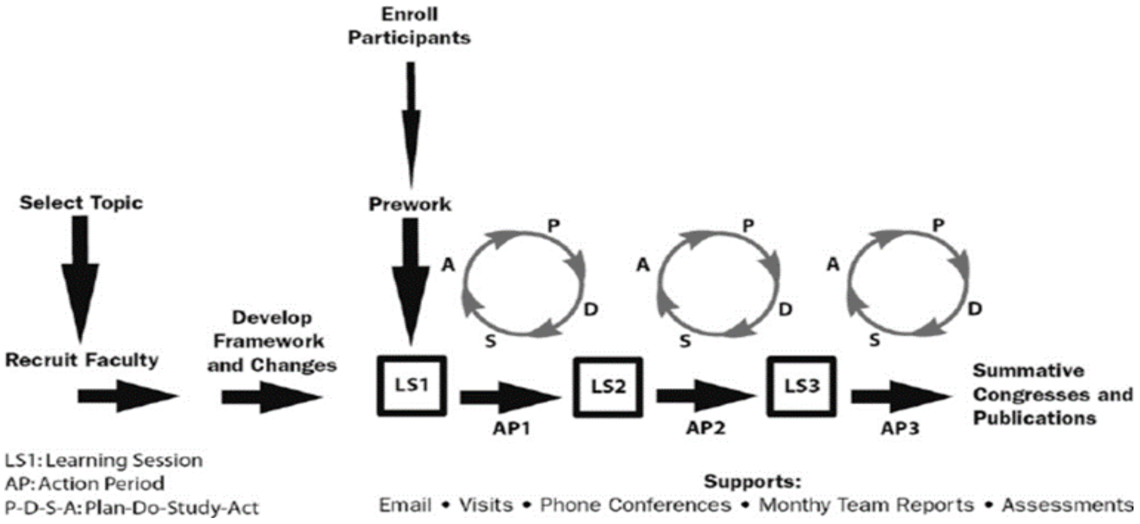

Figure 1 Visualisation of the Breakthrough Series Collaborative approach

Each learning session provides guidance and instruction in the theory and practice of improving performance and exchange in the specific topic area and functions as a milestone along each country hubs' own individual path to improvement—with each team reporting on their methods and results, collectively reflecting on lessons learned, and providing social support and encouragement for making further changes. Country hubs receive the benefit of direct access to each other and senior experts in the field at these meetings, as well as through regular conference calls, online dialogue and frequent written updates.

The topic for each SHIRIM cycle will be selected in consultation with the Share-Net country hubs. Each country hub will be requested to think about urgent SRHR topics ready for developing and testing knowledge translation strategies.

The selected topic should meet the following selection criteria:

1) it is important that current policy and practice deviates from best available scientific knowledge;
2) there should be evidence describing the gap between research, policy and practice;
3) examples of better performance should exist and it should be believed that this can be done in other settings as well;
4) if possible, it is preferred that all country hubs work on a similar or closely related topic, like for instance gender-based violence or child marriage;
5) identified and recognised by Share-Net country hub steering committee as a priority area and willingness of members to work on this topic.

After the selection of the topic, the Share-Net International Model for Rapid Improvement requires the country knowledge hub teams to answer three questions.

Supplement to: Meijers K, van der Kwaak A, Aqel I, et al. Using a rapid knowledge translation approach for better sexual and reproductive health and rights in Bangladesh, Burundi, Indonesia, and Jordan. *Glob Health Sci Pract*. 2022;10(2): e2100461. https://doi.org/10.9745/GHSP-D-21-00461

FUNDAMENTAL QUESTIONS FOR IMPROVEMENT:

1. *What are we trying to accomplish? (Aim)*

   Here, participants determine which specific outcomes they are trying to change through their work.

2. *How will we know that a change is an improvement? (Measures)*

   Here, team members identify appropriate measures to track their success.

3. *What changes can we make that will result in improvement? (Changes)*

   Here, teams identify key changes that they will actually test.

Key changes are implemented in a cyclical fashion (figure 1). Teams thoroughly **plan** to test and develop strategies (change package) that contribute to successful knowledge translation and use, taking into account cultural and organisational characteristics. Subsequently they **do** the work to make the change in their standard procedures, tracking their progress using quantitative measures. Next, they closely **study** the results of their work for insight on how to do better. Lastly, they **act** to make the successful changes permanent or to adjust the changes that need more work. This process continues serially over time and refinement is added with each cycle. These phases are known as “Plan-Do-Study-Act” (PDSA) cycles of learning (figure 1). Each PDSA cycle will include regular conference calls, online dialogue and frequent written updates.

Each country hub will establish a local collaborative team consisting of local stakeholders to transfer the knowledge gained through the international collaborative team and to test and develop strategies that contribute to successful knowledge translation and use. This will insure sustainability and localisation of the SHIRIM methodology and will engage the country level stakeholders in the development and implementation of the approach, which will guarantee ownership of the results.

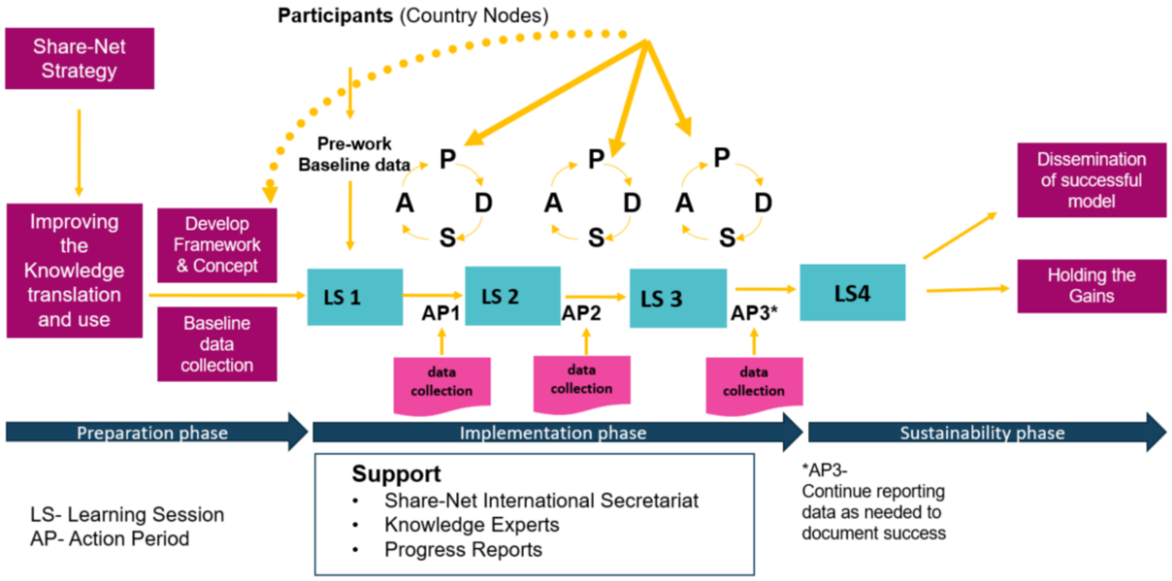

Figure 2 Visualisation of SHIRIM

Supplement to: Meijers K, van der Kwaak A, Aqel I, et al. Using a rapid knowledge translation approach for better sexual and reproductive health and rights in Bangladesh, Burundi, Indonesia, and Jordan. *Glob Health Sci Pract*. 2022;10(2): e2100461. https://doi.org/10.9745/GHSP-D-21-00461

PREPARATION PHASE

The preparation phase includes writing the SHIRIM collaborative concept, establishing the faculty for the local collaborative team, assigning the collaborative project manager, appointing the collaborative chair, and recruiting the knowledge experts according to the following steps:

- Establish the collaborative faculty (The collaborative chair, project manager, and knowledge experts)
    - Appoint the project manager. Role and responsibilities for the project manager include:
        - Write vacancy for content & knowledge experts
        - Organize brainstorm with country hubs on topic selection
        - Organize expert meeting with staff & content experts
        - Set up communication & support system with input from team
        - Organize and plan learning sessions 1-4
        - Write evaluation report
    - Appoint the chair. The role and responsibilities of the chair include:
        - Lead the knowledge experts team
        - Provide technical expertise and advise on the methodology of CRIM-KT
        - Take part in writing and reviewing the scientific article on the outcomes of CRIM-KT
        - Review literature and develop the technical material for the learning sessions
    - Appoint knowledge experts. Role and responsibilities of the knowledge experts include:
        - Teach and educate the collaborative teams of the country platforms during the learning sessions about specific technical topics such as:
            - Approaches to knowledge sharing and translation
            - Innovative knowledge translation tools
            - Presentation of state-of-the-art knowledge for selected SRHR topics
            - Best practices in knowledge use
        - Develop of an outcome measurement system
        - Assist country knowledge platform teams in designing the change package for each implementation period
        - Work as a mentor for the teams during the implementation period
        - Document the best impact (results) of implanting different change packages and share with the country knowledge platform teams during the third learning session.
        - Brainstorm with country knowledge platform teams on topic selection
        - Set up communication and M&E reporting system

IMPLEMENTATION PHASE:

The implementation phase included conducting three international learning sessions and applying the gained knowledge through three action periods as follows:

Learning Session 1: Share-Net International Rapid Improvement Model & Capitalisation Workshop

- Country knowledge platforms prepare and present story board with ideas, opportunities and challenges
- The Share-Net International Rapid Improvement Model for knowledge translation and use is explained by the collaborative chair.

Supplement to: Meijers K, van der Kwaak A, Aqel I, et al. Using a rapid knowledge translation approach for better sexual and reproductive health and rights in Bangladesh, Burundi, Indonesia, and Jordan. *Glob Health Sci Pract*. 2022;10(2): e2100461. https://doi.org/10.9745/GHSP-D-21-00461

- PDSA Cycle is explained
- Topic brainstorm session is organised to narrow the focus and scope
- Specific focus and scope within the overall topic is decided
- Senior leader for each country platform is appointed
- Capitalisation workshop is organised
- First change package is developed
- Each country knowledge platform makes a planning for the first implementation period

Action Periods

During each learning cycle the country platforms test agreed strategies from the change package together with their local stakeholders. Key changes are implemented in a cyclical fashion. This process continues serially over time and refinement is added with each cycle; these are known as PDSA cycles of learning.

- Country platforms facilitate local learning sessions for their stakeholders
- Change package is developed during the international learning sessions and is reviewed, updated and further developed by the local stakeholders during the local learning sessions
- Share-Net International supports the local country teams in their improvement work
- Collaboration and shared learning between the country platforms takes place
- Knowledge experts assess collaboration and progress
- Country platforms use each other as resources
- Conference calls are scheduled with the knowledge platform teams to update each other on progress, challenges, opportunities and lessons learned

Learning session 2 & 3: Country platforms learned and exchanged best practices in knowledge translation

- Problems and challenges are presented by the country platforms which are identified during the implementation periods that need to be addressed before the next action period, including problems with faculty and technical content
- Country platform story boards are presented explaining the strategies used and what worked and what didn't work in the previous action period
- Brainstorm session are organised on the next steps
- Expert workshop 'Best practices in knowledge translation on child marriage and teenage pregnancy' is facilitated by the knowledge experts
- Change package for learning session 2 and 3 are developed by the country knowledge platforms
- Each country knowledge platform makes a planning with concrete actions for the following implementation period
- Multi-Stakeholder Partnerships are set-up if applicable within the country context

Supplement to: Meijers K, van der Kwaak A, Aqel I, et al. Using a rapid knowledge translation approach for better sexual and reproductive health and rights in Bangladesh, Burundi, Indonesia, and Jordan. *Glob Health Sci Pract*. 2022;10(2): e2100461. https://doi.org/10.9745/GHSP-D-21-00461

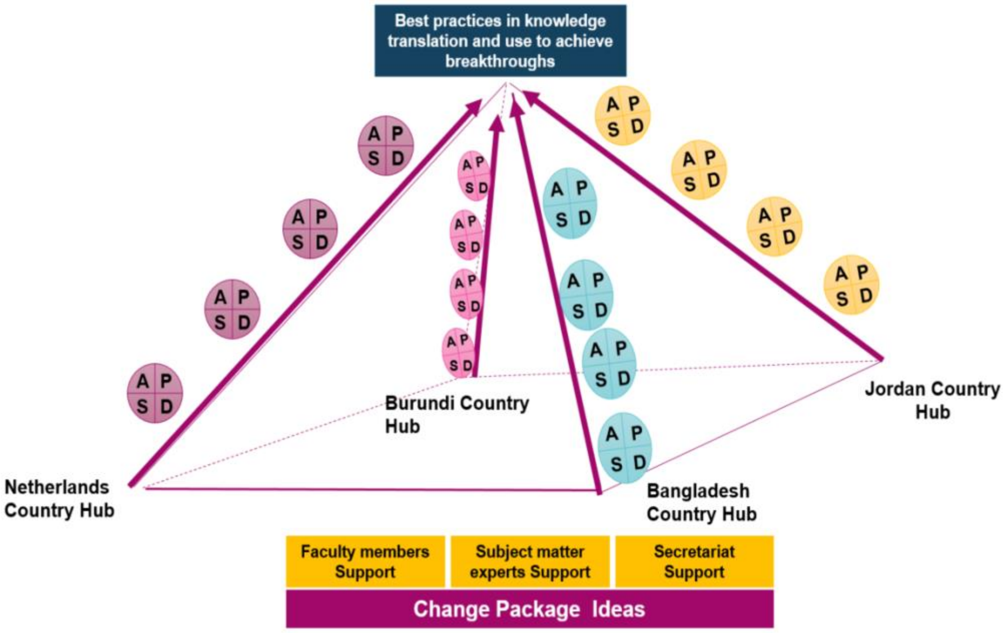

Figure 3 Visualisation of knowledge exchange between country hubs

SUSTAINABILITY PHASE:

Learning session 4: Sustaining successful strategies and establishing multi-stakeholders' partnerships

- Country platform story boards are presented that explain the strategies developed and shared experiences of what worked, what didn't work and it is discussed why.
- Best strategies for knowledge transfer are presented and documented for each country knowledge platform that show promising results
- Write-shop to draft success stories on how the CRIM-KT approach has helped in improving (changing) practice and policy in their country
- Multi-Stakeholder Partnerships are set up if possible within the country context

Communication & Reporting System

Regular progress updates will be written by the country hubs followed by conference calls which will keep track of progress and identify challenges. Content experts will assist in addressing these. After each implementation period a progress report will be written by the country hubs for monitoring and evaluation. The knowledge products they developed will be shared.

The outputs of the documentation and reporting will include:

- Country Hubs Storyboards
- Country hubs Change Packages
- Learning sessions materials
- Learning sessions reports

Supplement to: Meijers K, van der Kwaak A, Aqel I, et al. Using a rapid knowledge translation approach for better sexual and reproductive health and rights in Bangladesh, Burundi, Indonesia, and Jordan. *Glob Health Sci Pract*. 2022;10(2): e2100461. https://doi.org/10.9745/GHSP-D-21-00461

- Minutes of conference calls during the implementation periods

Outcomes

Each of the country hubs will develop specific knowledge products based on their expertise with support from the content experts and the secretariat of Share-Net International. In addition to knowledge products, SNI will write a scientific article that presents the approach and process and link it to the outcomes.

5. TIMEFRAME

Proposed duration: 15 months (possible extension to 18 months)

YEAR															
Activity/Month	O	N	D	J	F	M	A	M	J	J	A	S	O	N	D
Preparation Phase															
**Learning Session 1** Collaborative Approach & Capitalisation Workshop in Amsterdam															
**Implementation Period 1** Plan-Do-Study-Act															
**Learning Session 2** Country Hub Meeting before the annual business meeting															
**Implementation Period 2** Plan-Do-Study-Act															
**Learning Session 3** Country Hub Meeting & Mid-term Review															
**Implementation Period 3** Plan-Do-Study-Act															
**Learning Session 4** Summative Meeting Country Hubs Article Write-shop															

Supplement to: Meijers K, van der Kwaak A, Aqel I, et al. Using a rapid knowledge translation approach for better sexual and reproductive health and rights in Bangladesh, Burundi, Indonesia, and Jordan. *Glob Health Sci Pract*. 2022;10(2): e2100461. https://doi.org/10.9745/GHSP-D-21-00461

Progress Report Writing															
Conference Call															
Country Hubs Articles & Evaluation Report															
