# Supplementary material for: Using a Rapid Knowledge Translation Approach for Better Sexual and Reproductive Health and Rights in Bangladesh, Burundi, Indonesia, and Jordan
Source: Glob Health Sci Pract. 2022 Apr 28;10(2):e2100461. doi: 10.9745/GHSP-D-21-00461 (PMC9053141; doi:10.9745/GHSP-D-21-00461)

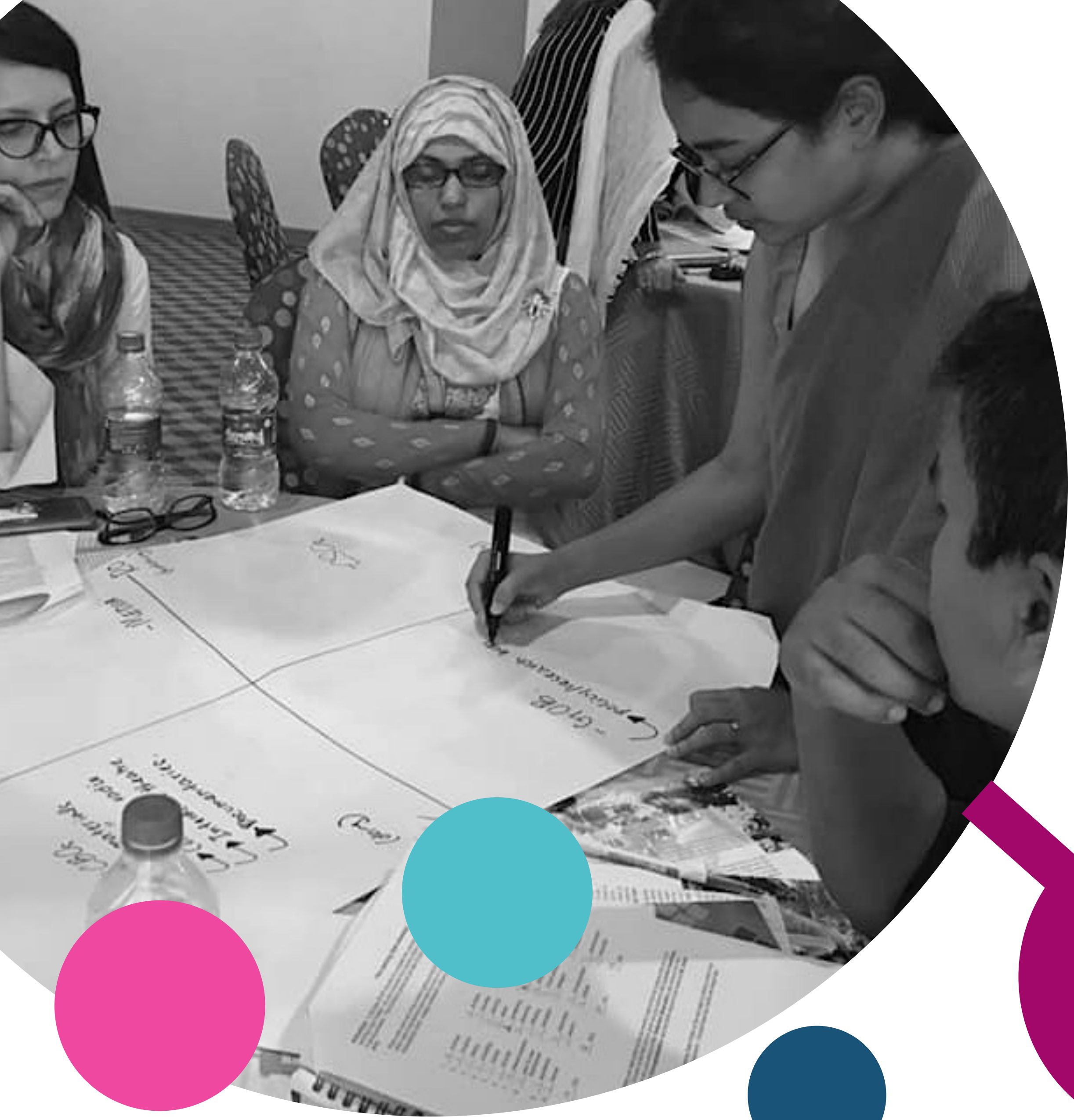

Share-Net Bangladesh
The Knowledge Platform on Sexual and Reproductive Health & Rights

Share-Net International Rapid Improvement Model (SHIRIM) for Knowledge Translation

Hosted by: RedOrange Media and Communications

www.share-netbangladesh.org

Opportunities

There are opportunities for effective collaboration between policy makers, researchers and practitioners in which country specific knowledge is generated, shared, translated and used, so that better informed programmes and policies addressing child marriage and/or teen age pregnancy are developed.

Problem Statements

Main Stakeholders Related Problem Statement:

Absence of organised and structured knowledge on the consequences of Child Marriage and the violation of rights and related responses by NGOs and CSOs.

Country Specific Problem Statement:

There is a lack of coordination and interaction between different policy makers, researchers and practitioners who are working in Bangladesh resulting in limited knowledge sharing and translation on effective interventions to address child marriage.

Aim of Collaborative Approach in Bangladesh

Sharing and applying knowledge about the most effective strategies that contribute to successful knowledge gathering, translation and sharing on child marriage issues in Bangladesh.

Learning session 1

Key drivers* (	Change Ideas** (concepts)	Specific Actions *** PDSA	Responsibility	Output Measure	Outcome Measure
Mapping of child marriage activities	Developed an information hub of organisations working on child marriage	– Develop format for the information collection<br>– Share with possible stakeholders<br>– Collect information<br>– Collate information categorically<br>– Validate<br>– Add menu bar on the website<br>– Upload on the website	– Share-Net Bangladesh Team<br>– All the participants	– Specific space has been created on Share-Net Bangladesh website and information are being shared on the platform	– Information hub is ready for use by the stakeholders for further collaboration

Key driver 1: Mapping of child marriage activities

Collaborative Approach on Child Marriage in Bangladesh

Organisation name: Plan International Bangladesh, The Bangladesh National Women Lawyers' Association (BNWLA)

Project Name: PHR (Protecting Human Rights)

Learning session 2

Key drivers*	Change Ideas** (concepts)	Specific Actions *** PDSA	Responsibility	Output Measure	Outcome Measure
Sharing best practices	A best practice "case study book"	– Case study collection by interviewing project directors/managers<br>– Copy editing<br>– Case validation<br>– Designing the book	Core team and home team	- 300 copies of case study book will be printed and disseminated<br>- e-copy will be disseminated through SNBD platform	- Knowledge is shared on best practices of CM projects in Bangladesh<br>- Increased knowledge addressing CM in Bangladesh

Key driver 2: Highlighted of Initiatives Addressing Child Marriage in Bangladesh

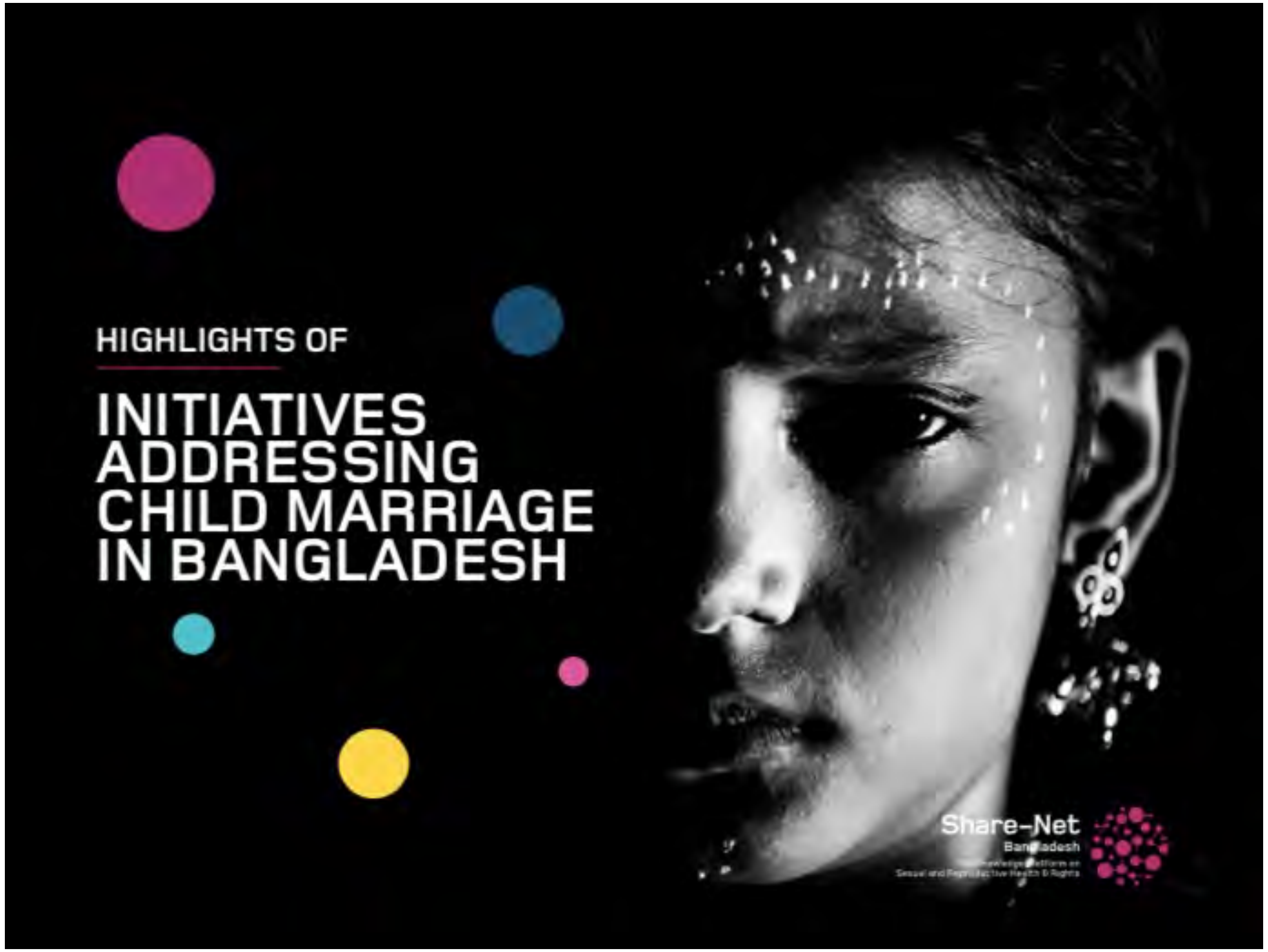

Learning session 3

Key drivers*	Change Ideas** (concepts)	Specific Actions *** PDSA	Responsibility	Output Measure	Outcome Measure
Collaboration among stakeholders for strengthening the advocacy effort	Organising round-table discussion to share the lessons learnt in promoting the CM issue in Bangladesh	– Identifying partners<br>– Developing concept note<br>– Inviting guests<br>– Organising round-table meeting<br>– Sharing the outcome on SNBD website	Core team and home team	- 20/25 participants join the meeting from 18 organisations	- Knowledge is translated and shared<br>- Increased collaboration among partners

Key driver 3: Strengthening the collaboration among stakeholders ensuring coordinated effort to address Child Marriage in Bangladesh

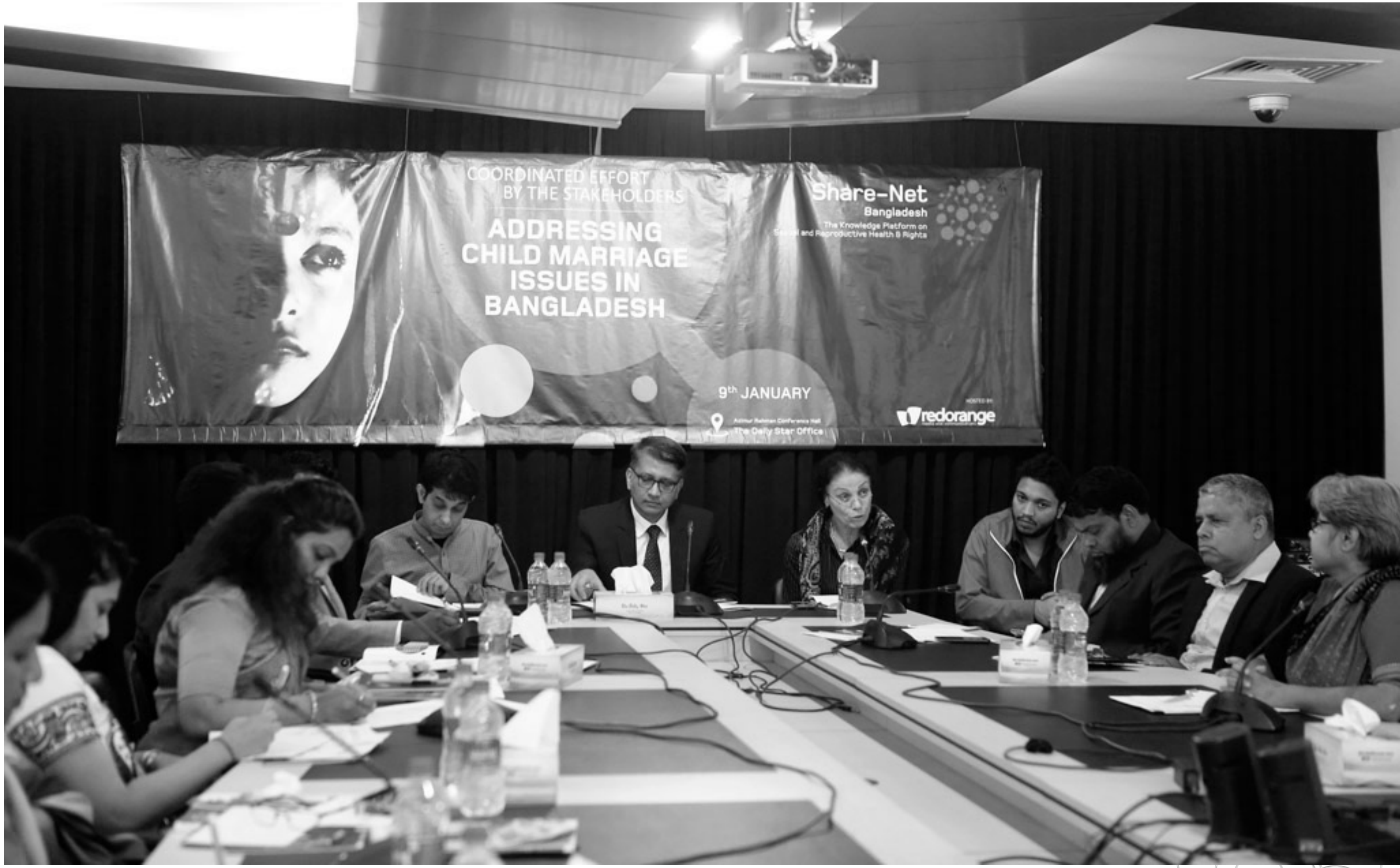

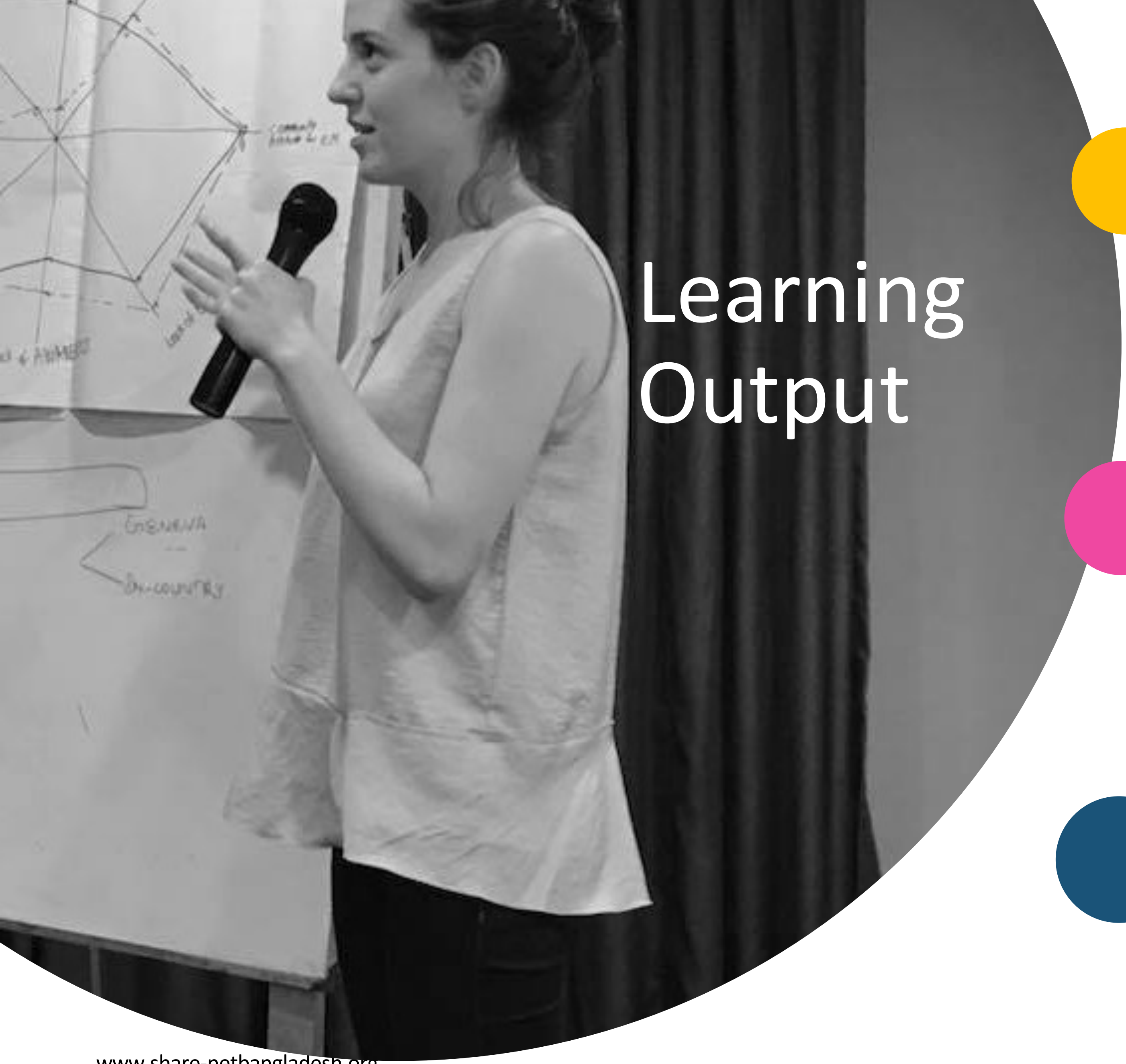

Learning Output

Output of the 1st learning session

Created an information hub of organisations working on child marriage in Bangladesh

Output of the 2nd learning session

Highlighted of Initiatives Addressing Child Marriage in Bangladesh

Output of the 3rd learning session

Roundtable meeting organised: Strengthening the collaboration among stakeholders ensuring coordinated effort to address Child Marriage in Bangladesh

Main Challenges

- Bringing all the stakeholders on time
- Commitment from the participants
- Difficulties in getting their appointments
- Budget

Lessons Learnt

Organising the learning session for a day

Involve members to take some of the sessions

THANK YOU

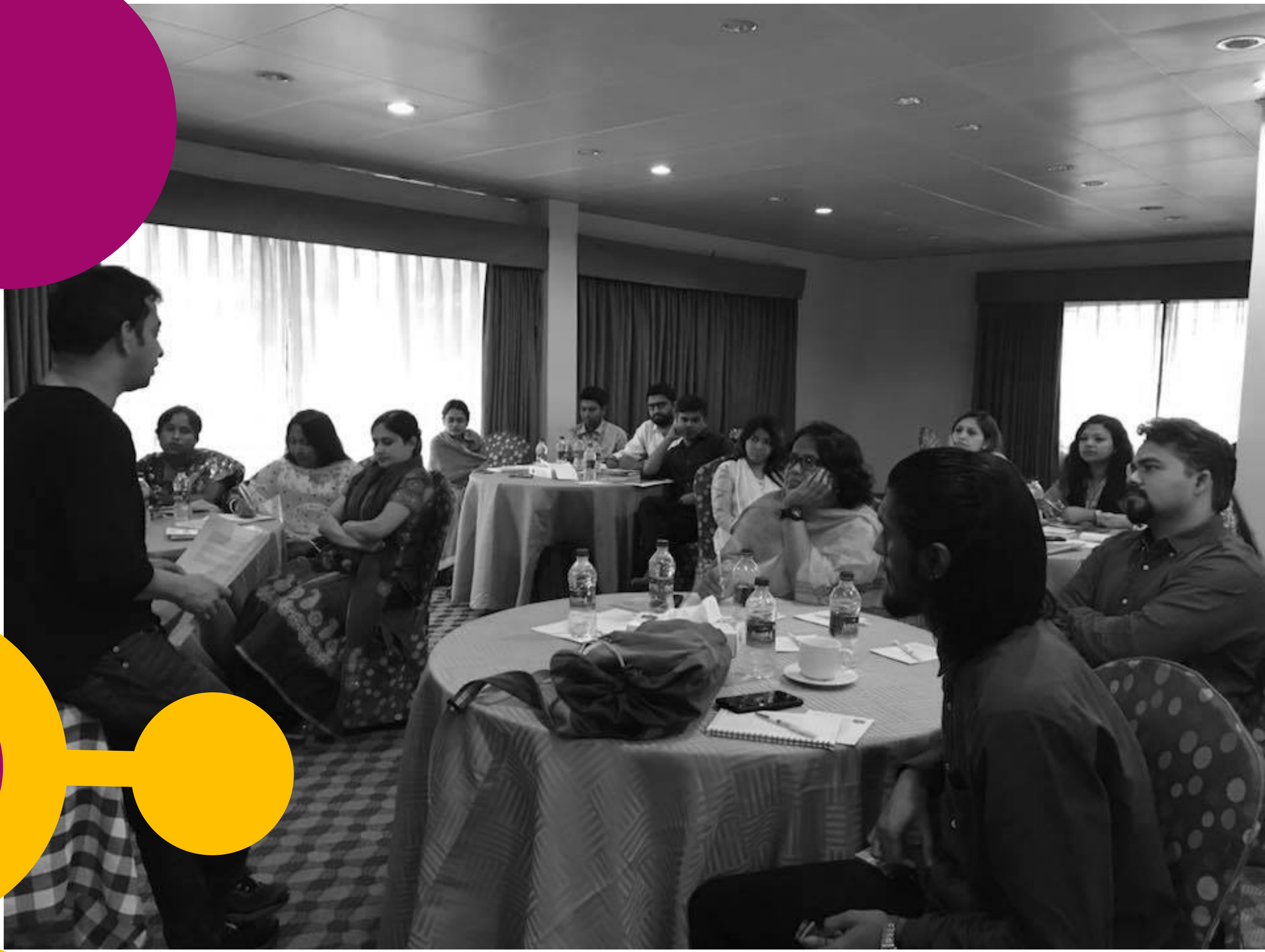

Share-Net International
The Knowledge Platform on
Sexual and Reproductive Health & Rights

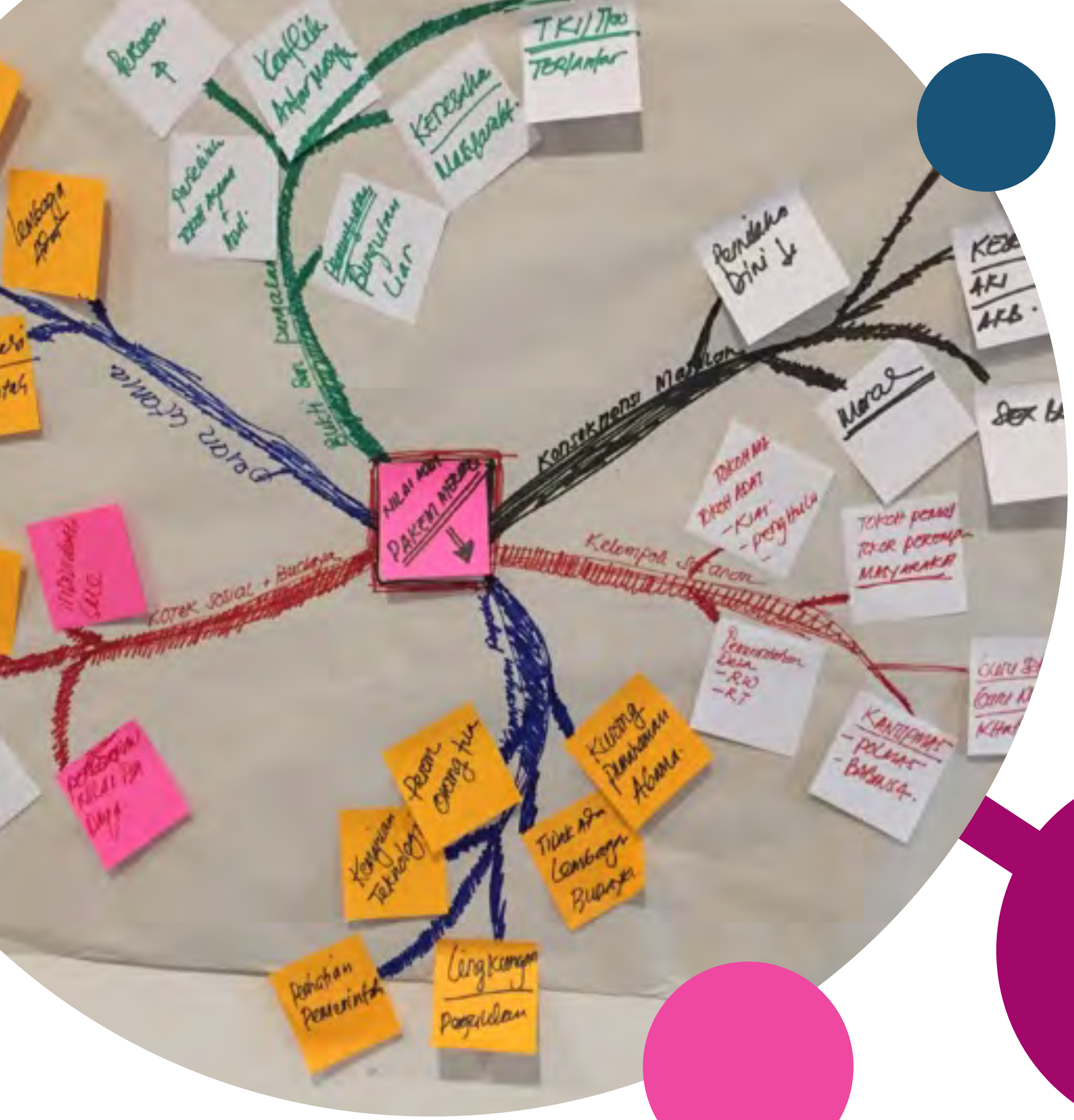

Share-Net

The Netherlands-Indonesia

SHIRIM
Learning session 1 - 3

www.share-netinternational.org

Share-Net International Rapid Improvement Model (SHIRIM) for Knowledge Translation The Collaborative Approach

Rutgers WPF Indonesia

Share-Net Netherlands – Indonesia Collaborative

About Rutgers WPF Indonesia

Rutgers WPF Indonesia is a centre of expertise organization on reproductive health, sexuality and the prevention of sexual and gender-based violence. Rutgers WPF Indonesia develops programs to improve young people's access towards comprehensive sexual education and youth-friendly services, as well as encourages men's engagement to prevent gender-based violence. Rutgers WPF Indonesia works together with partners in the local level. The organization has proven its track record in the related fields and its innovations in the policy advocacy, research, gender-transformative approach and supporting local partners. Rutgers WPF Indonesia works with more than 18 partners in 13 provinces around Indonesia.

Local team

Rutgers Indonesia

1. Amala Rahmah
2. Tia Fitriyanti
3. Ely Sawitri

IPPF West Lombok

1. Ahmad Hidayat
2. Aryan Agus Pratama

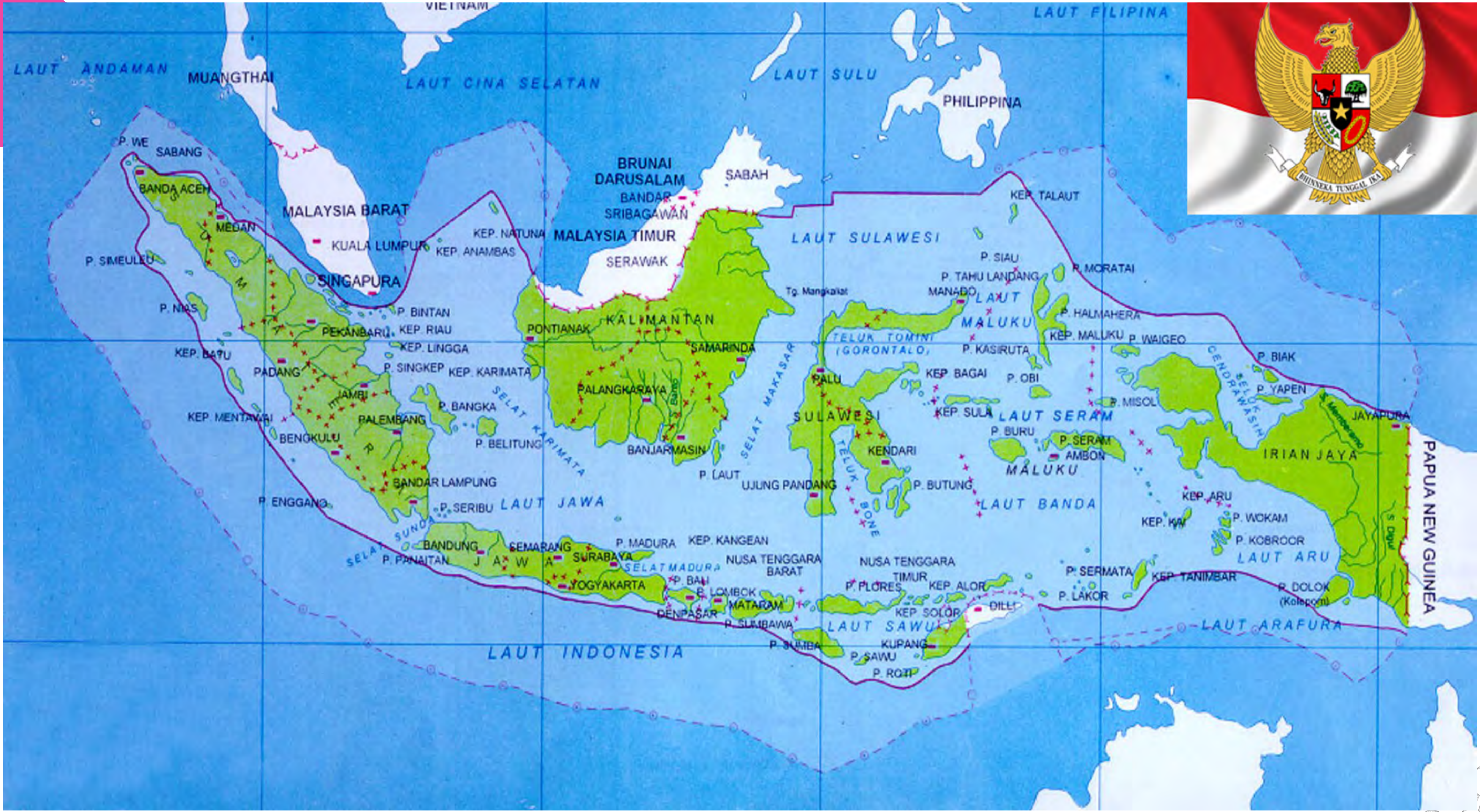
LAUT ANDAMAN
MUANGTHAI
LAUT CINA SELATAN
LAUT SULU
PHILIPPINA
LAUT FILIPINA
BRUNAI DARUSALAM
SABAH
MALAYSIA BARAT
MALAYSIA TIMUR
SERAWAK
KUALA LUMPUR
SINGAPURA
KALIMANTAN
LAUT SULAWESI
SULAWESI
LAUT MALUKU
LAUT SERAM
MALUKU
LAUT BANDA
IRIAN JAYA
PAPUA NEW GUINEA
LAUT JAWA
LAUT INDONESIA
LAUT SAWU
LAUT ARAFURA
LAUT ARU
BANDA ACEH
MEDAN
PADANG
PEKANBARU
JAMBI
PALEMBANG
BENGKULU
BANDAR LAMPUNG
PONTIANAK
PALANGKARAYA
BANJARMASIN
SAMARINDA
MANADO
PALU
KENDARI
UJUNG PANDANG
AMBON
JAYAPURA
BANDUNG
SEMARANG
SURABAYA
YOGYAKARTA
DENPASAR
MATARAM
KUPANG
DILLI
NUSA TENGGARA BARAT
NUSA TENGGARA TIMUR

Child Marriage and Teenage Pregnancy Data in Indonesia

Percentage of ever-married women aged 20 to 24 married before age 18, 16 and 15, 2008 to 2015

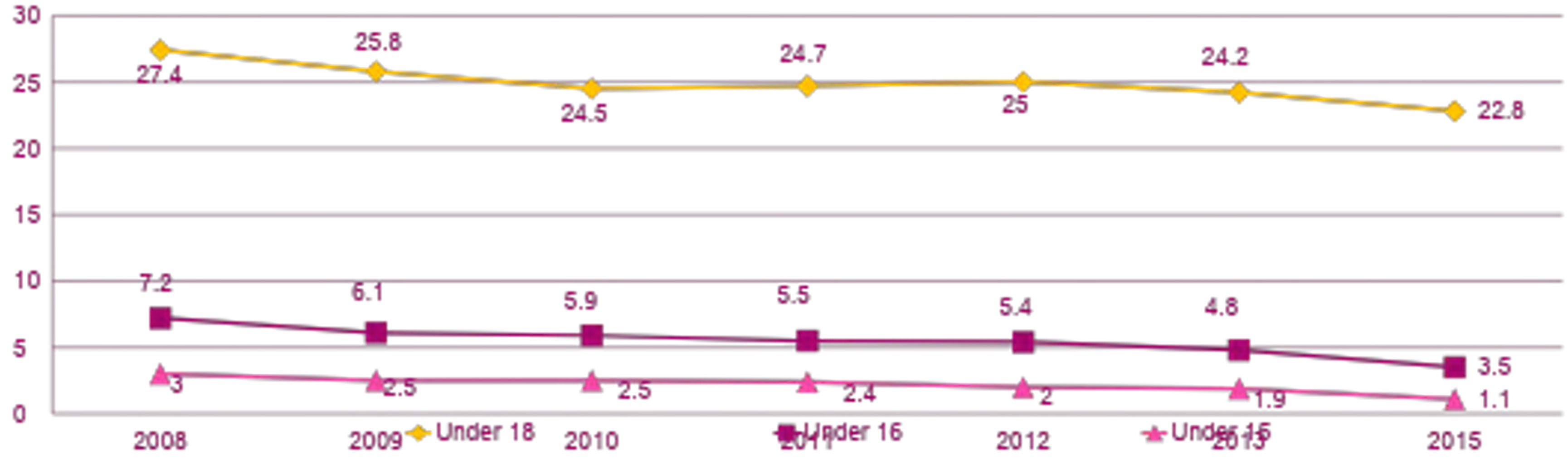

The child marriage rate in Indonesia remains high, with over one in four girls currently marrying before reaching adulthood. Furthermore, the rate has plateaued in recent years.

(Source: Secondary analysis of SUSENAS 2008-2012, 2013 and 2015)

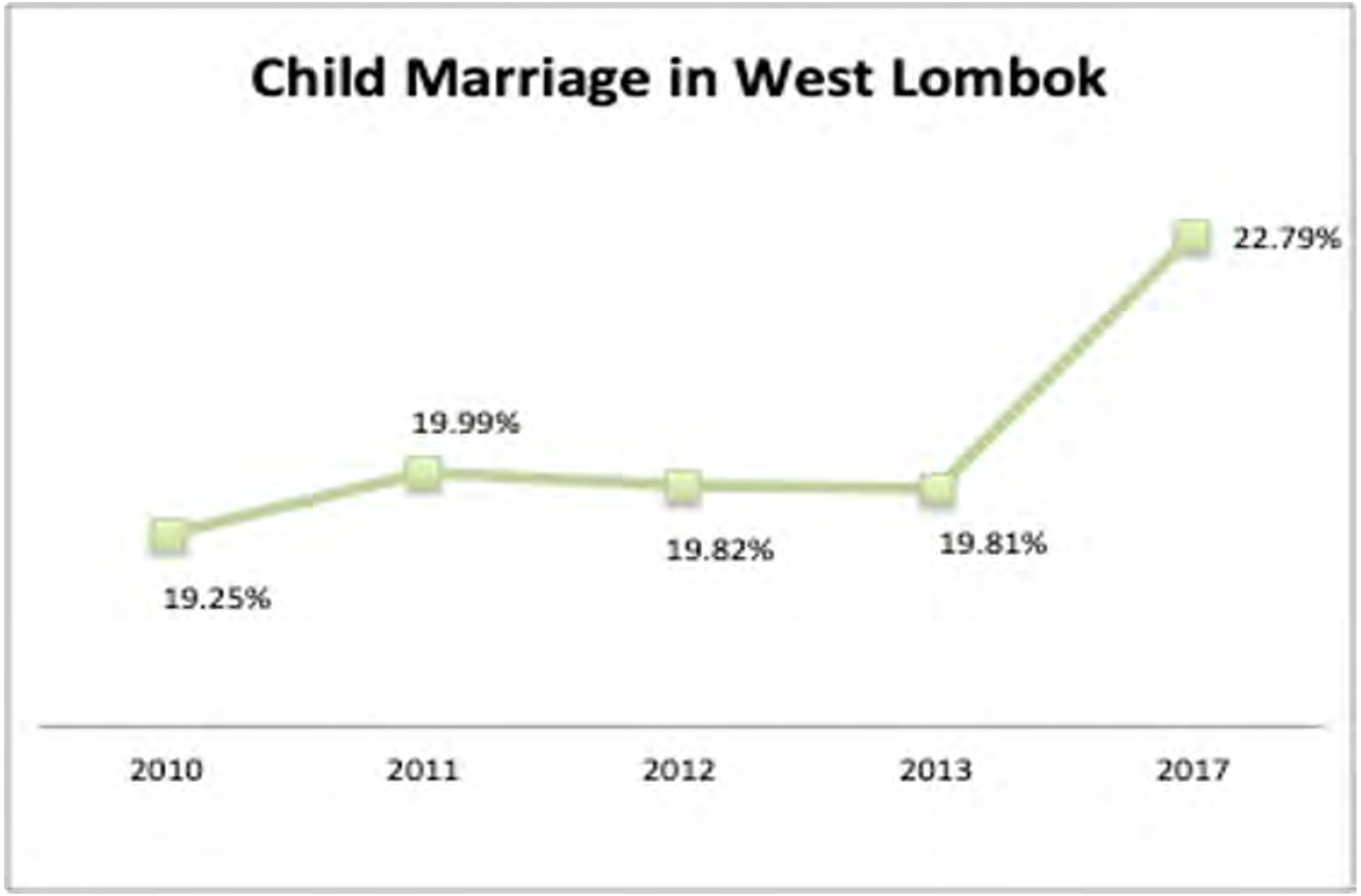

Source: District of Population, Family Planning Service, Women's Empowerment and Child Protection Office

Problem Evidence

1. UNICEF INDONESIA: Research brief, Child Marriage In Indonesia: Progress On Pause. 2016
2. Badan Pusat Statistik (Central Bureau of Statistics): Progress On Pause: Child Marriage Data Analysis in Indonesia. 2016
3. The Meaning Of Merarik And Nyongkolan For Bridal Couple In West Nusa Tenggara. A study by Febri Triwahyudi, Achmad Mujab Masykur. 2014
4. Merarik Marriage Based On Prescriptive Law Of Sasak Lombok Tribe-West Nusa Tenggara. A study by RAHAYU LIANA, SH. 2006
5. Baseline Yes I Do in West Lombok. 2016
6. Child Marriage in West Lombok Research by Ford Foundation and Rumah Kitab. 2015
7. Operational Research on Divorce for Yes I Do Program. 2017

Problem statement

Misinterpretation of Merarik Culture that contributes to high incidence of child marriage in West Lombok District.

Aim

Increase *knowledge and practice* of the relevant stakeholders, especially village heads of *Merarik guideline* to reduce high number of child marriage in West Lombok

Main relevant stakeholders

1. **District Health Office,**
2. **District Education Office,**
3. **Local NGOs,**
4. **Board of Art,**
5. **Religious Leaders,**
6. **Traditional Leaders,**
7. **University representatives**
8. **District of Population, Family Planning Service, Women's Empowerment and Child Protection Office**

Local Collaborative LS1

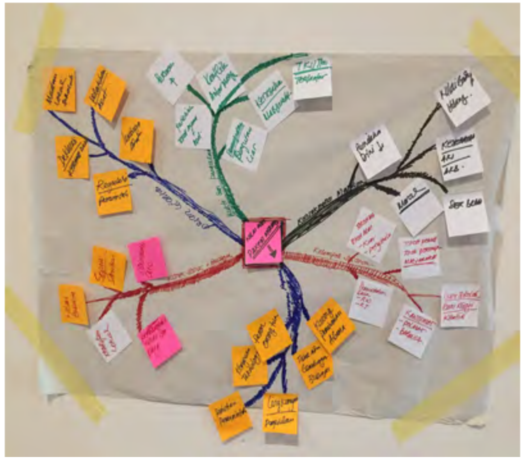

- On 8 January 2018: Learning session 1 participated by District Working Group. Identifying the fundamental causes of increasing number of child marriage in Lombok Barat by using the information methods (radar diagram, problem tree and mind mapping).
- On 9 January 2018: Develop guideline on Merarik Culture
- Participants: District Health Office, District Education Office, District Family Planning Service, Women's Empowerment and Child Protection Office, Local NGOs (Gagas, IPPF West Lombok), Board of Art, Religious Leaders, Traditional Leaders, University representatives, and youth led organization.

Country Hub Change package LS 1

Key drivers*	Change Ideas** (concepts)	Specific Actions *** PDSA	Responsibility	Output Measure	Outcome Measure
**Knowledge Generation**	Sharing knowledge of stakeholders on "Merarik" Culture related to child marriage	Series of focus grup discussions with key influencers, policy makers, NGO's , researchers, parents, young people.	Country Team	- Number (5) of focus group discussions conducted<br>- Number of stakeholder participated (15)	Information collected from different stakeholder and summarized for sharing
**Stakeholders involvement (Engagement)**	Common understanding between stakeholder on "Merarik" Culture related to child marriage	Round table meeting for all groups/stakeholders	Core and Country Team	- Number (2) of round table conducted<br>- Number of stakeholder participated (15)<br>- (2) Meeting reports	Developed action plan for each institution with defined roles and responsibilities

Local change package of LS 1

Key drivers*	Change Ideas** (concepts)	Specific Actions *** PDSA	Responsibility	Output Measure	Outcome Measure
**Involving traditional and religious leaders**	Similarity of perception on Merariq culture among all traditional and religious leaders	Meeting with traditional and religious leaders	Traditional leaders associations	- Number of meeting conducted (1)<br>- Number of leaders participated (15)	Common understanding between traditional leaders and religious leaders on Merarik Culture
**Stakeholders involvement (Engagement)**	Common understanding between stakeholder on Merarik Culture	Workshop to develop guideline on Merarik Culture	District Working Group (DWG) West Lombok	- Number of workshop conducted (1)<br>- Number of stakeholder participated (15)<br>- Number of reports (1)	Guideline on Merarik Culture

Local Collaborative LS 2

- On 23 April 2018: Learning session 2 was attended by the District Working group. Topic: Reflection on the development process and finalization of the draft of the Merariq guidelines compiled by the local team. Methods used: VAK Learning style and Kolb experiential learning theory and Margolis wheel.
- On 24 April: Launching and Dissemination of the Merarik guideline by the District Head.
- Participants: District Health Office, District Education Office, District of Population, Family Planning Service, Women's Empowerment and Child Protection Office, Local NGOs (Gagas, PKBI), Board of Art, Religious Leaders, Traditional Leaders, University representatives, and youth led organization, representatives of District Office, Women and Child protection Center, village heads and authorities (100+)

Country Hub Change package LS 2

Key drivers*	Change Ideas** (concepts/results)	Specific Actions *** PDSA	Responsibility	Output Measure	Outcome Measure
**Communication and coordination between stakeholders**<br><br>**15 April**	Final guidelines on Merariq	Roundtable with DWG (district) + all traditional and religious leaders of West Lombok	Local team (Rutgers, PKBI, DWG)	- Final Guideline on Merarik Culture (Involving youth in this process)<br>- Number of meeting conducted (1)<br>- Number of leaders participated (15)	Stakeholders are agreed the guideline (YID activity/budget)
**Stakeholders involvement (Engagement)**<br><br>**Learning Session 2**<br><br>**Day 1. Learning session with DWG member**<br><br>**Day 2. Dissemination guidelines + action plan formulation to sub-district government + traditional and religious leaders**	Participants: government sub-district level + religious + trad leaders)	Day 1 (23 April): learning/sharing of experiences + new techniques learning (DWG district)<br><br>Day 2 (24 April): 0,5 day dissemination plan (Participants: government sub-district level + religious + trad leaders)	District Working Group (DWG) West Lombok + stakeholders	Guidelines disseminated + further dissemination plan (for village level) developed<br><br>- Number of workshop conducted (1)<br>- Number of stakeholder participated (16)<br>- Number of reports (1)<br><br>- Number of stakeholder participated (100)	Stakeholders are committed to implement the Merarik guidelines

Local change package of LS 2

Stakeholders involvement (Engagement)	**Development of the syllabus for training of Merariq guideline**	**Action period: August 2018**	**District working group**	- **Action period report (1)**<br>- **Syllabus of the training (1)**<br>- **Number of stakeholders (5)**	**Stakeholders are committed to conduct and involve in the training of Merariq guideline as facilitator**
	Develop training syllabus (1 day)<br><br>Merarik guidelines trained to the head of the villages	Action period: August 2018	Head of the village (4 villages)	- Action period report (1)<br>- Number of villages government staff participated (16)	Village government staffs understand and follow the Merarik guidelines. This will decrease of the approval for young couple under 18 yo from the head of villages.
	Monitoring of the merarik guideline implementation	Action period: September-December 2018	District working group	- Monitoring report (1)<br>- Number of stakeholders participated (16)	Stakeholders are committed to implement and monitor of the merarik guideline implementation.

Local Collaborative LS 3

- On 8 January 2019: Learning session 3 was participated by the District Working group. Agenda: Review the scope, mandate, roles and responsibilities for each stakeholder (Population and Family Planning Office, Health Office, Education Office, Religion Office, etc) to ensure sustainability of the implementation Guidelines.
- On 21-22 November 2018. Training/dissemination to 40 village heads.

Country Hub Change package LS 3

Key drivers*	Change Ideas** (concepts/results)	Specific Actions *** PDSA	Responsibility	Output Measure	Outcome Measure
**Stakeholders engagement**	Development of the syllabus for training of Merarik guideline (0,5 day)	Action period:<br>October 2018	Committee of District working group	- Action period report (1)<br>- Syllabus of the training (1)<br>- Number of stakeholders (5)	Stakeholders are committed to conduct and involve in the training of Merarik guideline as facilitator
	Training of Merariq guidelines to the head of the villages (1,5 day)<br>Piloting of the Merarik guideline Implementation by village head	Action period:<br>October 2018<br>Action period:<br>October-December 2018	· Village authorities<br>· Stakeholders in DWG	- Action period report (1)<br>- Number of heads of village participated (40)	Head of the villages understand and follow the Merarik guidelines.
	1. Learning session 3 (1 day)<br>2. Round table meeting of development of the agreement letter among the institutions of the DWG (ex District of Family planning) to adopt the guideline and allocating budget for village heads training in another villages (1 day)<br>(review the scope, mandate, Role and responsibility)	Action period:<br>November 2018	- DWG members	- Action period report (1)<br>- Number of DWG members (16)	
**Monitoring**	Monitoring of the merarik guideline implementation<br>(collecting success story)	Action period:<br>December 2018	District working group	- Monitoring report (1)<br>- Number of stakeholders participated	Stakeholders are committed to implement and monitor of the merarik guideline implementation.

The most successful:

- Commitment of the stakeholders (DWG) to be involved in each Learning Session and Implementing the Action Plan.
- Merarik guideline developed in English and Bahasa Indonesia.
- Activity and budget sharing allocation between Share Net and DWG to disseminate the merarik guideline (joint action).
- Merarik guideline is included in Technical Guidelines for Anti Child Marriage Movement (government regulation) to be applied in West Lombok.

To be followed up

- Monitoring of the Merarik guideline implementation

Knowledge Product

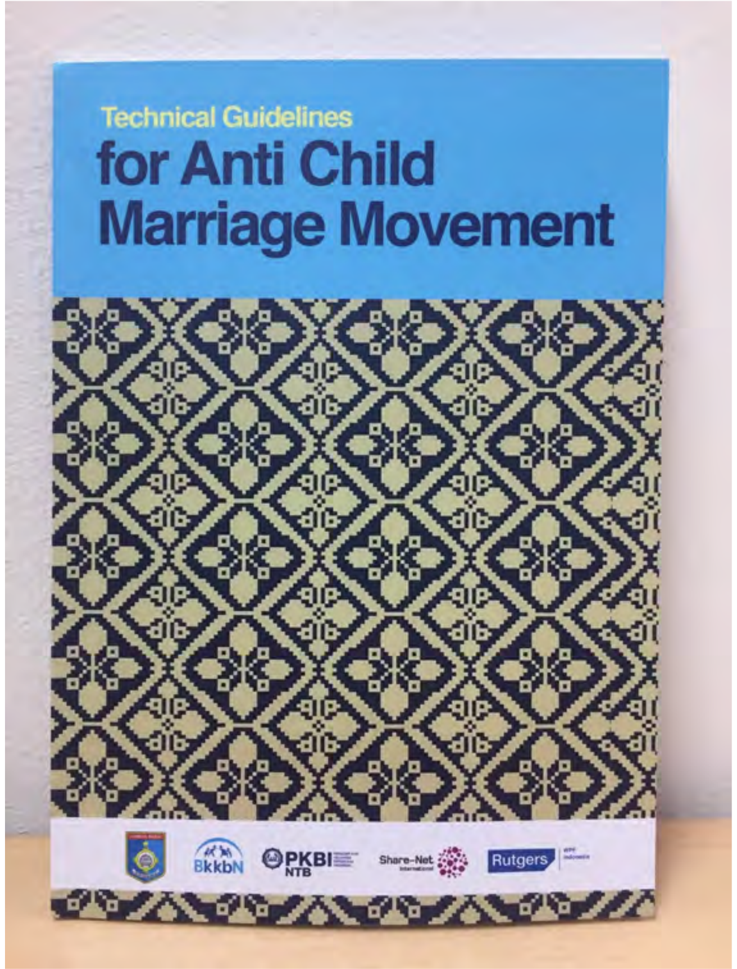

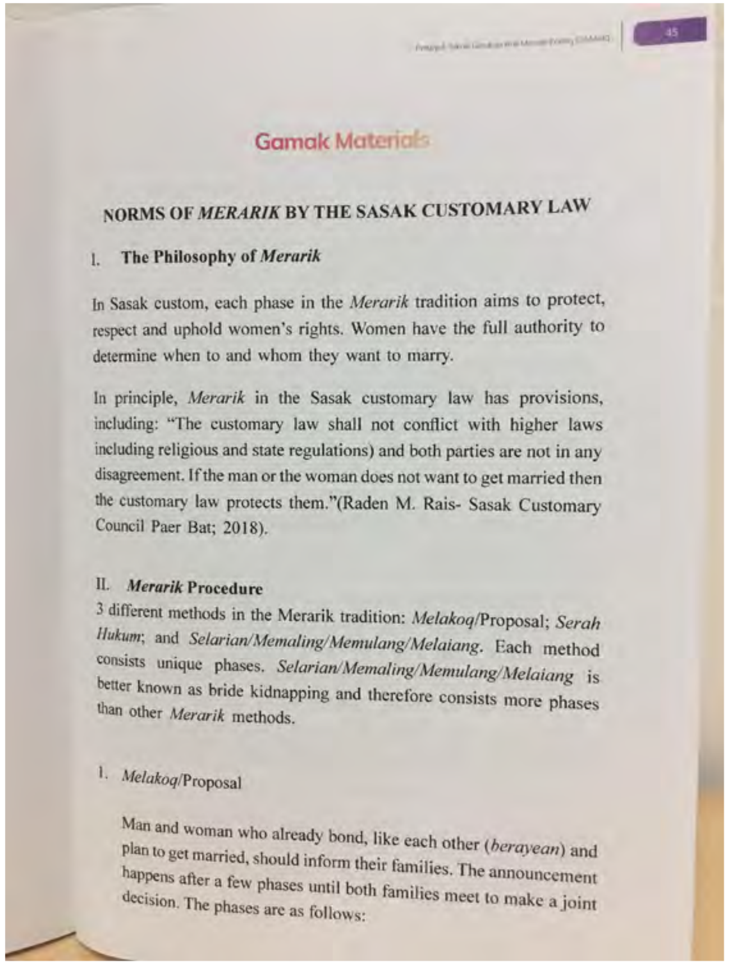
Gamak Materials

NORMS OF *MERARIK* BY THE SASAK CUSTOMARY LAW

I. **The Philosophy of *Merarik***

In Sasak custom, each phase in the *Merarik* tradition aims to protect, respect and uphold women's rights. Women have the full authority to determine when to and whom they want to marry.

In principle, *Merarik* in the Sasak customary law has provisions, including: "The customary law shall not conflict with higher laws including religious and state regulations) and both parties are not in any disagreement. If the man or the woman does not want to get married then the customary law protects them."(Raden M. Rais- Sasak Customary Council Paer Bat; 2018).

II. ***Merarik* Procedure**

3 different methods in the Merarik tradition: *Melakoq*/Proposal; *Serah Hukum*; and *Selarian/Memaling/Memulang/Melaiang*. Each method consists unique phases. *Selarian/Memaling/Memulang/Melaiang* is better known as bride kidnapping and therefore consists more phases than other *Merarik* methods.

1. *Melakoq*/Proposal

Man and woman who already bond, like each other (*berayean*) and plan to get married, should inform their families. The announcement happens after a few phases until both families meet to make a joint decision. The phases are as follows:

Outcomes and Impacts of Knowledge Product

Outcome :

Village heads apply Merarik culture according to true Sasak Customary Law

Impact :

Decreasing child marriage caused by misinterpretation of Merarik Culture in West Lombok

Multi-stakeholders Partnership

- Yes I Do Country and Local Alliance – Monitoring implementation of Merarik Guidelines + Expand socialization of Merarik Guideline
- District Working Group – Developing Belas (Customary Law to Postpone Marriage and Monitoring implementation of Merarik Guidelines)
- Adolescent Network of CM Prevention – Advocate for increase marriage age at national level
- Girls Not Brides Network in Indonesia – Visit of Princess Mabel, Publish Case Stories

A people without the knowledge of their past history, origin and culture is like a tree without roots

-Marcus Garvey-

THANK YOU

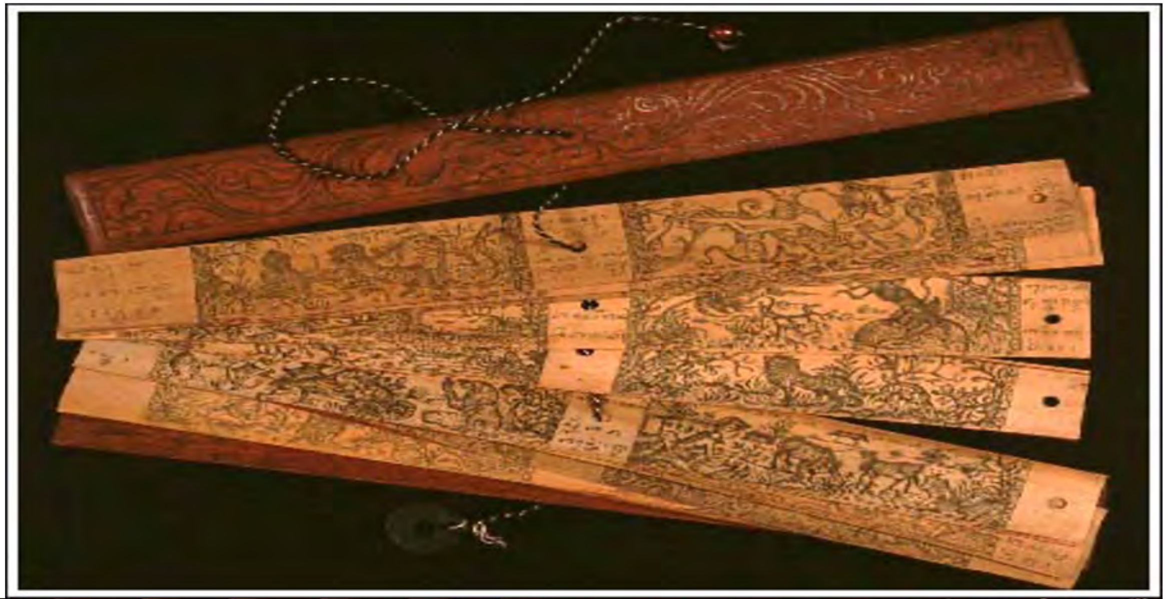

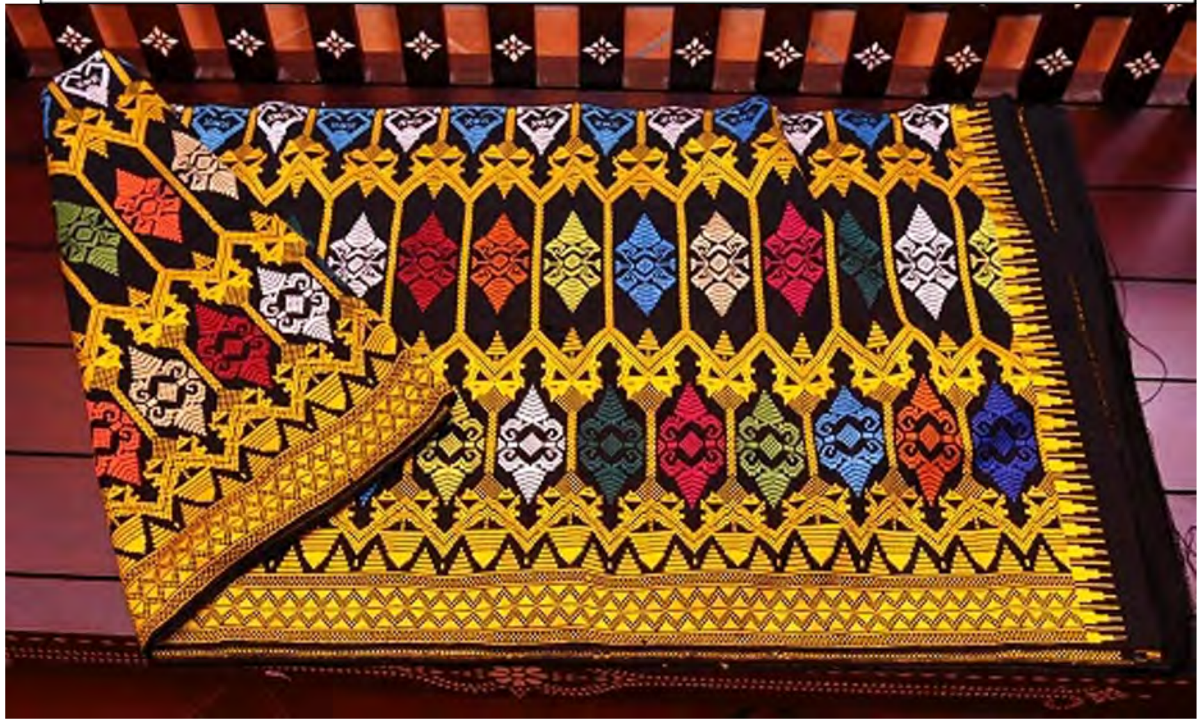

Supplement: GHSP-D-21-00461-Supplement2.pdf [file GHSP-D-21-00461-Supplement2.pdf]
