# Supplementary material for: Using a Rapid Knowledge Translation Approach for Better Sexual and Reproductive Health and Rights in Bangladesh, Burundi, Indonesia, and Jordan
Source: Glob Health Sci Pract. 2022 Apr 28;10(2):e2100461. doi: 10.9745/GHSP-D-21-00461 (PMC9053141; doi:10.9745/GHSP-D-21-00461)
Supplement: GHSP-D-21-00461-Supplement1-4-5.pdf [file GHSP-D-21-00461-Supplement1-4-5.pdf]

Supplement to: Meijers K, van der Kwaak A, Aqel I, et al. Using a rapid knowledge translation approach for better sexual and reproductive health and rights in Bangladesh, Burundi, Indonesia, and Jordan. *Glob Health Sci Pract*. 2022;10(2): e2100461. https://doi.org/10.9745/GHSP-D-21-00461

Supplement 1. Share-Net International Rapid Improvement Model as a phased approach

Preparation Phase

The preparation phase includes writing the CRIM-KT concept note, establishing the faculty for the local knowledge platform team, assigning the collaborative project manager, appointing the collaborative chair, and recruiting the knowledge experts according to the following steps:

- Establish the collaborative team (chair, project manager and knowledge experts)
    - Appoint the project manager. Role and responsibilities for the project manager include:
        - Write vacancy for content & knowledge experts
        - Organize brainstorm with country hubs on topic selection
        - Organize expert meeting with staff & content experts
        - Set up communication & support system with input from team
        - Organize and plan learning sessions 1-4
        - Write evaluation report
    - Appoint the chair. The role and responsibilities of the chair include:
        - Lead the knowledge experts team
        - Provide technical expertise and advise on the methodology of CRIM-KT
        - Take part in writing and reviewing the scientific article on the outcomes of CRIM-KT
        - Review literature and develop the technical material for the learning sessions
    - Appoint knowledge experts. Role and responsibilities of the knowledge experts include:
        - Teach and educate the collaborative teams of the country platforms during the learning sessions about specific technical topics such as:
            - Approaches to knowledge sharing and translation
            - Innovative knowledge translation tools
            - Presentation of state-of-the-art knowledge for selected SRHR topics
            - Best practices in knowledge use
        - Develop of an outcome measurement system
        - Assist country knowledge platform teams in designing the change package for each implementation period
        - Work as a mentor for the teams during the implementation period
        - Document the best impact (results) of implanting different change packages and share with the country knowledge platform teams during the third learning session.
        - Brainstorm with country knowledge platform teams on topic selection
        - Set up communication and M&E reporting system

Implementation Phase:

The implementation phase included conducting three international learning sessions and applying the gained knowledge through three action periods as follows:

Learning Session 1: Share-Net International Rapid Improvement Model & Capitalisation Workshop

- Country knowledge platforms prepare and present story board with ideas, opportunities and challenges
- The Share-Net International Rapid Improvement Model for knowledge translation and use is explained by the collaborative chair.
- PDSA Cycle is explained
- Topic brainstorm session is organised to narrow the focus and scope
- Specific focus and scope within the overall topic is decided
- Senior leader for each country platform is appointed
- Capitalisation workshop is organised
- First change package is developed
- Each country knowledge platform makes a planning for the first implementation period

Action Periods

During each learning cycle the country platforms test agreed strategies from the change package together with their local stakeholders. Key changes are implemented in a cyclical fashion. This process continues serially over time and refinement is added with each cycle; these are known as PDSA cycles of learning.

- Country platforms facilitate local learning sessions for their stakeholders
- Change package is developed during the international learning sessions and is reviewed, updated and further developed by the local stakeholders during the local learning sessions
- Share-Net International supports the local country teams in their improvement work
- Collaboration and shared learning between the country platforms takes place
- Knowledge experts assess collaboration and progress
- Country platforms use each other as resources
- Conference calls are held with the knowledge platform teams to update each other on progress, challenges, opportunities and lessons learned

Learning session 2 & 3: Country platforms learned and exchanged best practices in knowledge translation

- Problems and challenges are presented by the country platforms which are identified during the implementation periods that need to be addressed before the next action period, including problems with faculty and technical content
- Country platform story boards are presented explaining the strategies used and what worked and what didn’t work in the previous implementation period
- Brainstorm session are organised on the next steps
- Expert workshop ‘Best practices in knowledge translation on child marriage and teenage pregnancy‘ is facilitated by the knowledge experts

- Change package for learning session 2 and 3 are developed by the country knowledge platforms
- Each country knowledge platform makes time planning with concrete actions for the following implementation period
- Multi-Stakeholder Partnerships are set-up if applicable within the country context

Sustainability Phase:

Learning session 4: Sustaining successful strategies and establishing multi-stakeholders' partnerships

- Country platform story boards are presented that explain the strategies developed and shared experiences of what worked, what didn't work and it is discussed why.
- Best strategies for knowledge transfer are presented and documented for each country knowledge platform that show promising results
- Write-shop to draft success stories on how the CRIM-KT approach has supported improving practice and policy in their country
- Multi-Stakeholder Partnerships are set up if possible within the country context

Supplement to: Meijers K, van der Kwaak A, Aqel I, et al. Using a rapid knowledge translation approach for better sexual and reproductive health and rights in Bangladesh, Burundi, Indonesia, and Jordan. *Glob Health Sci Pract*. 2022;10(2): e2100461. https://doi.org/10.9745/GHSP-D-21-00461

SUPPLEMENT 4. AGENDAS CRIM-KT INTERNATIONAL LEARNING SESSIONS

Agenda CRIM-KT Learning Session 1

Date: 8th – 10th of November 2017

Place: Amsterdam, the Netherlands

Day 1: Wednesday, 8th of November 2017

Time	Duration	Activity
9:00 – 9:20	20 Minutes	Walk-in and Storyboard Set-up
9:20 – 9:35	15 Minutes	Welcoming remarks<br>The CRIM-KT mission and goals in relation to the Share-Net International 5-year Strategy
9:35 – 9:45	10 Minutes	Overview Learning Sessions 1-4<br>CRIM-KT trajectory period: October 2017 – December 2018<br>Learning session 1 agenda and arrangements
9:45 – 10:30	40 Minutes	Plenary & Group Discussion:<br>The Collaborative Approach as a model for structured learning and improvement
10.30 – 11.00	30 Minutes	Group Exercise:<br>The Peg Game to explain CRIM-KT and illustrate the importance of working together, documenting progress, avoiding to make the same mistakes and repeating successful approaches
11:00 – 11:30	30 Minutes	Break
11:30 – 12:10	40 Minutes	Plenary:<br>Methods and tools for analyzing root causes, organizing and presenting information for each of the following tools:<br>1. Flow Chart<br>2. Fishbone Diagram<br>3. Tree Diagram<br>4. Interrelationship Diagram<br>5. Radar Chart
12.10 – 12.45	35 Minutes	Group Work:<br>Use one method to analyze root causes and organizing

		information on teenagepregnancy or child narriage for your country knowledge platform
12.45 – 13.45	60 Minutes	Lunch
13.45 – 14.30	45 Minutes	Plenary Presentations & Discussion<br>5 min. presentation & 5 min. discussion per country platform
14:30 – 15:00	30 Minutes	Plenary:<br>The Rapid Improvement Model PDSA cycle (Plan, Do, Study, Act)
15:00 – 15:30	30 Minutes	Group Exercise:<br>Through a Sequence exercise the country platforms learn how to test, re-test and adapt their assumptions through adopting the PDSA-cycle
15:30 – 16.00	30 Minutes	Q&A and update about agenda day 2
16.00 – 16.30	30 Minutes	Group Work:<br>Prepare story board presentation

Supplement to: Meijers K, van der Kwaak A, Aqel I, et al. Using a rapid knowledge translation approach for better sexual and reproductive health and rights in Bangladesh, Burundi, Indonesia, and Jordan. *Glob Health Sci Pract*. 2022;10(2): e2100461. https://doi.org/10.9745/GHSP-D-21-00461

Day 2: Thursday, 9th of November 2017

Time	Duration	Activity
8.45 – 9.00	15 Minutes	Walk-inn
9.00 – 9.10	10 Minutes	Opening day 2 & planning
9:10 – 9:30	20 Minutes	Plenary & Discussion:<br>World best practices in reducing child marriage & teenage pregnancy
9:30 – 9:35	5 Minutes	Plenary & Questions:<br>Discussion best practices child marriage & teenage pregnancy
9:35 – 10.35	60 Minutes	Plenary Presentation & Feedback:<br>Story boards tour, country knowledge platform presentations<br>10 min. presentations and 5 min discussion for each country
10:35 – 11:00	25 Minutes	Plenary:<br>Technical lecture on capitalization, knowledge generation, sharing, use and transfer.<br>Instruction mind mapping of the Collaborative Problem
11:00 – 11:30	30 Minutes	Break
11:30 – 12.30	60 Minutes	Group Work:<br>Mind mapping<br>Every country knowledge platform made their own mind map, with their problem statement in the center.<br><br>Aim: To unravel a key issue and explore all its aspects in a creative way
12:30 – 13.10	40 Minutes	Plenary Presentation & Feedback:<br>Mind Mapping Tour<br>Country platforms present their mind map<br>5 min. presentation and 5 min. discussion
13.10 – 14.10	60 Minutes	Lunch
14.10 – 14.40	30 Minutes	Group Work:<br>Update Story Board

14:40 -15:00	20 Minutes	Plenary Presentation:<br>5 min. presentation per country hub
15:00 – 15:45	45 Minutes	Plenary & Discussion:<br>Explaining the change package template
15:45 – 16:30	45 Minutes	Group Work:<br>Brainstorm on change package
16:30 – 17:00	30 Minutes	Q&A and update about agenda day 2

Day 3: Friday, 10th of November 2017

Time	Duration	Activity
8.15 – 8.30	15 Minutes	Walk-in
8.30 – 8.40	10 Minutes	Opening<br>Planning day 2
8.40 – 9.25	45 Minutes	Group Work:<br>Finalize change packages
9.25 – 10:05	40 Minutes	2 Presentations<br>4 min. presentation per country knowledge platform and 3 min. discussion
10:05 – 10:35	30 Minutes	Break
10.35 – 10.55	20 Minutes	2 Presentations<br>4 min. presentation per country knowledge platform and 3 min. discussion
10.55 – 11.50	55 Minutes	Plenary:<br>Documentation and reporting<br>CRIM-KT communication system
11:50 – 12.30	40 Minutes	Group work:<br>Adapt change package incorporating input from discussion<br>Make list of potential partners for local core team<br>Make planning & budget for action period 1
12.30 – 12.45	15 Minutes	Plenary:<br>Instruction budget
12.45 – 13.30	45 Minutes	Lunch
13.30 – 14.30	60 Minutes	Plenary Presentations & Discussion:<br>5 min. presentation + 10 min. discussion per country knowledge platform
14.30 – 15.30	60 Minutes	Group work:<br>Draft agenda for local learning session 1<br>Determine which content of the international learning sessions is suitable and/or requires adaptation to be used in the local context.

15.30- 15.45	15 Minutes	Break
15.45 – 16.45	60 Minutes	Practical work:<br>Practice facilitating next local learning session
16.45 – 16.55	10 Minutes	Q&A<br>Local learning sessions organized by country knowledge platforms
16.55 – 17.00	5 Minutes	Closing remarks

Supplement to: Meijers K, van der Kwaak A, Aqel I, et al. Using a rapid knowledge translation approach for better sexual and reproductive health and rights in Bangladesh, Burundi, Indonesia, and Jordan. *Glob Health Sci Pract*. 2022;10(2): e2100461. https://doi.org/10.9745/GHSP-D-21-00461

Agenda CRIM-KT Learning Session 2

Date: 21-23 March 2018

Place: Amsterdam, the Netherlands

Day 1: 21 March 2018

Time	Duration	Activity
8:45 – 9:15	30 Minutes	Storyboard set-up
9:15 – 9:25	10 Minutes	Welcoming remarks
9:25 – 9:40	15 Minutes	Overview LS2 agenda and arrangements
9:40 – 10:00	20 Minutes	Story board gallery setting up to exchange experiences
10:00 –11:00	60 Minutes	Story board gallery walk<br>Each country hub member will present the country storyboard for 15 minutes and 15 minutes for in depth discussion<br><br>Topics covered<br><br>How did they manage organizing and facilitating their first learning session? When was it?<br><br>Who have attended? No. of participants and which organizations?<br><br>Who did not attend? Why?<br><br>Did they presented storyboards?<br><br>Who presented/facilitated from the local team?<br><br>What was the most successful session in the LS1? Why?<br><br>Change package implementation progress<br><br>What activities were not implemented? Why?<br><br>What activities were implemented? How did it go? If you are doing it again, would you do it better? How?<br><br>What activities went well and you would like to keep or improve?<br><br>What activities didn't work well? Why? Are you keeping or replacing it with new ideas?
11:00 –11:20	20 Minutes	Break

Supplement to: Meijers K, van der Kwaak A, Aqel I, et al. Using a rapid knowledge translation approach for better sexual and reproductive health and rights in Bangladesh, Burundi, Indonesia, and Jordan. *Glob Health Sci Pract*. 2022;10(2): e2100461. https://doi.org/10.9745/GHSP-D-21-00461

11:20 –12:30	70 Minutes	Cont. story board gallery walk
12.30 –13.30	60 Minutes	Lunch

Supplement to: Meijers K, van der Kwaak A, Aqel I, et al. Using a rapid knowledge translation approach for better sexual and reproductive health and rights in Bangladesh, Burundi, Indonesia, and Jordan. *Glob Health Sci Pract*. 2022;10(2): e2100461. https://doi.org/10.9745/GHSP-D-21-00461

13:30 –<br>14:30	60 Minutes	Group activity:<br>Emphasize on strategies that worked and are successful.<br>How did the country knowledge platforms manage this? Exchange experiences<br>How to scale up their efforts?<br>How to use these strategies in other countries and settings? Will it work? If no, why?<br>What are appropriate next steps?
14:30 –<br>15:30	60 Minutes	Group discussion country knowledge platforms:<br>Challenges LS1 and AP1<br>Lessons learned LS1 and AP1<br>Similarities and differences between the country knowledge platforms
15:30 –<br>15:45	15 Minutes	Break
15:45 –<br>16:30	45 Minutes	Exercise and group discussion:<br>Introducing the VAK learning style
16:30 –<br>17:00	30 Minutes	Q&A and update about agenda day 2

Supplement to: Meijers K, van der Kwaak A, Aqel I, et al. Using a rapid knowledge translation approach for better sexual and reproductive health and rights in Bangladesh, Burundi, Indonesia, and Jordan. *Glob Health Sci Pract*. 2022;10(2): e2100461.
https://doi.org/10.9745/GHSP-D-21-00461

Day 2: 22 March 2018

Time	Duration	Activity
8:30 – 9:30	60 minutes	Reflection:<br>The Margolis wheel
9:30 – 10:30	60 Minutes	Plenary:<br>Updates about global best practices in reducing child marriage & teenage pregnancy
10:30 – 11:00	30 Minutes	Break
11:00 – 12:00	60 Minutes	Exercise and group discussion:<br>Using learning styles in designing knowledge translation activities<br><br>The Kolb learning style:<br>“Kolb's experiential learning theory works on two levels: a four-stage cycle of learning and four separate learning styles.”
12:00 – 12:30	30 Minutes	Plenary:<br>Best practices of knowledge translation and use including theoretical background<br><br>Practical exercise:<br>Work on knowledge translation & use
12:30 – 13:30	60 Minutes	Lunch
13:30 – 14:00	30 Minutes	Cont. Practical exercise:<br>Work on knowledge translation & use
14:00 – 15:00	60 Minutes	Plenary & exercise:<br>Ideation (idea generation tools)
15:00 – 16:00	60 Minutes	Plenary:<br>How to develop output and outcome measures<br>Theoretical session and practical exercise
16:00 – 16:15	15 Minutes	Break

16:15 – 17:00	45 Minutes	Plenary:<br>Presentation country knowledge platforms (10 minutes each):<br>New forms of knowledge generation<br><br>Ideation techniques<br><br>Output and outcome measures
17:00 – 17:30	30 Minutes	Q&A and update about agenda day 2

Day 3: 23 March 2018

Time	Duration	Activity
8:30 – 9:15	45 minutes	Reflection:<br>Networking cards activity
9:15 – 10:15	60 Minutes	Exercise and group discussion<br>Testing participants readiness to adopt change
10:15 – 10:30	15 Minutes	Break
10:30 – 11:50	80 Minutes	Group work:<br>Brainstorm on new ideas for AP2 change package<br>Practical work to adjust change package<br>What activities went well and would like to keep or improve?<br>What are the new ideas for change
11:50 – 12:30	40 Minutes	Plenary:<br>Presenting country knowledge platform change package
12:30 – 13:30	60 Minutes	Lunch
13:30 – 14.45	75 Minutes	Practical work:<br>Practice facilitating next local learning session
14:45 – 15:30	45 Minutes	Plenary:<br>M&E including reporting and the Collaborative communication system<br>Is communication with knowledge experts effective? Why not?<br>How we can improve it?
15:30 – 16:00	30 Minutes	Q&A about the country nodes local collaborative
16:00 – 16.15	15 Minutes	Evaluation learning session 2
16:15 – 16:30	15 Minutes	Closing remarks

Supplement to: Meijers K, van der Kwaak A, Aqel I, et al. Using a rapid knowledge translation approach for better sexual and reproductive health and rights in Bangladesh, Burundi, Indonesia, and Jordan. *Glob Health Sci Pract*. 2022;10(2): e2100461. https://doi.org/10.9745/GHSP-D-21-00461

Agenda CRIM-KT Learning Session 3

Date: 10 -12 September 2018

Place: Dhaka, Bangladesh

Day 1: 10 September 2018

Time	Duration	Activity
10:15 – 10.30	15 Minutes	Story board set-up<br>Bring memory stick
10:30 – 10:45	15 Minutes	Welcoming remarks<br>Overview (agenda and arrangements)<br>Round of introductions
10.45 – 11.00	15 Minutes	Icebreaker
11:00 – 12:00	60 Minutes	Story board gallery walk:<br>Each country hub member will present the country storyboard for 15 minutes and 15 minutes for in-depth discussion<br><br>Topics covered<br><br>How did they manage organizing and facilitating their second learning session?<br><br>When was it?<br><br>Who have attended? No. of participants? What organization they represent?<br><br>Who did not attend? Why?<br><br>Who presented/facilitated from the local team?<br><br>Did they present storyboards? Provide samples? Photos?<br><br>Was LS2 different from LS1? How?<br><br>What was new?<br><br>What was the most successful session in the LS2? Why?<br><br>Change package implementation progress<br><br>What activities were not implemented from the change package? Why? What should we do?

		What activities were implemented from the change package? How did it go? If you are doing it again, would you do it better? How?<br><br>What activities from the change package went well, and you would like to keep or improve?<br><br>What activities from the change package didn't work well? Why? Are you keeping or replacing it with new ideas?<br><br>Was there any new partnerships/joint projects or initiatives/joint activities established as a result of the SHIRIM? What is it? (Stories to tell about evidence of collaboration between stakeholders to address child marriage and teenage pregnancy)
12.00 – 13.00	60 Minutes	Lunch
13:00 – 14:00	60 Minutes	Cont. Story board gallery walk
14:00 – 15:00	60 Minutes	Group activity:<br>Emphasize on strategies that worked and are successful.<br><br>How to scale up successful initiatives/activities/strategies?<br><br>How to use these strategies in other countries and settings? Will it work? If no, why?<br><br>What are appropriate next steps?<br><br>Challenges from country hubs LS2 and AP2<br><br>Lessons learnt from country hubs LS2 and AP2
15.00 – 15.15	15 Minutes	Break
15.15 – 16.15	60 Minutes	Plenary:<br>Pitching ideas & 7 Tips from story telling method
16:15 – 17:00	45 Minutes	Group activity:<br>Approaches to manage conflict between stakeholders using the Thomas Kilmann conflict grid
17:00 – 17:10	10 Minutes	Q&A and update about agenda day 2

Supplement to: Meijers K, van der Kwaak A, Aqel I, et al. Using a rapid knowledge translation approach for better sexual and reproductive health and rights in Bangladesh, Burundi, Indonesia, and Jordan. *Glob Health Sci Pract*. 2022;10(2): e2100461.
https://doi.org/10.9745/GHSP-D-21-00461

Day 2: 11th of September 2018

Time	Duration	Activity
9:00 – 9:40	40 minutes	Exercise:<br>Johari Window Communication Model
9:40 – 10.10	30 Minutes	Plenary & Group Discussion<br>Literature review on teenage pregnancy
10:10 – 11:00	50 Minutes	Plenary<br>Stakeholders analysis and mapping
11:00 – 11:15	15 Minutes	Break
11:15 – 12:15	60 Minutes	Group activity:<br>Developing and presenting country Hubs stakeholders analysis and mapping
12:15 – 13:00	45 Minutes	Plenary:<br>Designing and facilitating Multi-stakeholders' Partnerships
13:00 – 14:00	60 Minutes	Lunch
14:00 – 14:40	40 Minutes	Group activity:<br>Brainstorming and presenting ideas about possible Multi-stakeholders' Partnerships
14:40 – 15:40	60 Minutes	Plenary and group discussion:<br>Fund raising methods and techniques for country knowledge platforms
15.40 – 16.00	20 Minutes	Plenary:<br>Policy Influencing & writing Policy Briefs
16:00 – 16:15	15 Minutes	Break
16:15 – 17:00	45 Minutes	Group activity:<br>Using FIRO Behavioral Style inventory to understand different stakeholders
17:00 – 17:10	10 Minutes	Q&A and update about agenda day 2

Supplement to: Meijers K, van der Kwaak A, Aqel I, et al. Using a rapid knowledge translation approach for better sexual and reproductive health and rights in Bangladesh, Burundi, Indonesia, and Jordan. *Glob Health Sci Pract*. 2022;10(2): e2100461.
https://doi.org/10.9745/GHSP-D-21-00461

Day 3: 12th of September 2018

Time	Duration	Activity
8:30 – 9:15	45 minutes	Reflection:<br>Drawing the future<br>Video: who moved my cheese
9:15 – 10:15	60 Minutes	Plenary & group work:<br>Writing success stories
10:15 – 10:30	15 Minutes	Break
10:30 – 11:30	60 Minutes	Group work:<br>Brainstorm on new ideas for AP3 change package<br><br>Practical work to adjust change package<br><br>What activities went well and would like to keep or improve?<br><br>What are the new ideas for change<br><br>Draft programme local LS3
11:30 – 13:00	90 Minutes	Plenary:<br>Country knowledge platforms present change package and practical work to practice facilitating next local learning session
13:00 – 14:00	60 Minutes	Lunch
14:00 – 14.45	45 Minutes	M&E including reporting and the collaborative communication system<br><br>Is communication with knowledge experts effective? Why not? How we can improve it?<br><br>Reporting via Google Drive
14:45 – 15:15	30 Minutes	Q&A about CRIM-KT at county knowledge platform level
15:15 – 15:30	15 Minutes	Closing remarks

Supplement to: Meijers K, van der Kwaak A, Aqel I, et al. Using a rapid knowledge translation approach for better sexual and reproductive health and rights in Bangladesh, Burundi, Indonesia, and Jordan. *Glob Health Sci Pract*. 2022;10(2): e2100461.
https://doi.org/10.9745/GHSP-D-21-00461

Agenda CRIM-KT Learning Session 4

Date: 14-16 January 2019

Place: Death Sea & Amman, Jordan

Day 1: 14th of January 2019

Time	Duration	Activity
8:30 – 8:45	15 Minutes	Story board set-up
8:45 – 9:00	15 Minutes	Welcoming remarks<br>Overview (agenda and arrangements)
9:00 – 10:30	90 Minutes	Story boards<br>Each country hub member will present the country storyboard for 20 minutes and 15 minutes for in depth discussion<br><br>Topics covered<br><br>Problem statement, statistics and evidence<br><br>Change packages implementation progress from LS1 to LS4<br><br>What activities were implemented successfully from the change packages? How did it go? If you are doing it again, would you do it better? How?<br><br>What activities from the change package didn't work well? Why?<br><br>What knowledge products were developed? Present samples (pictures)<br><br>What impacts and outcomes of knowledge products were measured<br><br>Were there any new Multi-stakeholders 'partnerships / joint projects or initiatives/joint activities established as a result of the SHIRIM? What is it? (Stories to tell about evidence of collaboration between stakeholders to address child marriage and teenage pregnancy)
10:30 – 11:00	30 Minutes	Break
11:00 – 13:00	120 Minutes	Cont. story boards
13.00 – 14.00	60 Minutes	Lunch

14:00 – 15:00	60 Minutes	Plenary & Exercise:<br>Documenting success stories
15:00 – 16:00	60 Minutes	Group activity:<br>Emphasize on strategies that worked and are successful. (Holding Gains)<br><br>How to scale up successful initiatives/activities/strategies?<br><br>How to use these strategies in other countries and settings? Will it work? If no, why?<br><br>Lessons learnt from country hubs
16:00 – 16.15	15 Minutes	Break
16:15 – 17.00	45 Minutes	Group activity:<br>The change readiness assessment
17:00 – 18:00	60 Minutes	Evaluation:<br>Interviews with the knowledge platform representatives

Day 2: 15th of January 2019

Time	Duration	Activity
8.45 – 9.00	15 Minutes	Icebreaker
9:00 – 9:40	40 Minutes	Knowledge Products Fair:<br>Showcasing developed knowledge products
9:40 – 10.00	60 Minutes	Plenary:<br>SHIRIM evaluation
10:00 – 11:00	60 Minutes	Plenary:<br>Fundraising & proposal writing
11:00 – 12:00	60 Minutes	Break
12:00 – 13:00	60 Minutes	Group activity:<br>Using the “Change Curve” in knowledge translation
12:45 – 13:45	60 Minutes	Lunch
13:45 – 14:45	60 Minutes	Group activity:<br>Color Brain Communication CBCT<br>Using colors to communicate with stakeholders

Supplement to: Meijers K, van der Kwaak A, Aqel I, et al. Using a rapid knowledge translation approach for better sexual and reproductive health and rights in Bangladesh, Burundi, Indonesia, and Jordan. *Glob Health Sci Pract*. 2022;10(2): e2100461. https://doi.org/10.9745/GHSP-D-21-00461

14.45 – 15.45	30 Minutes	Plenary and group discussion
15:45 – 16:00	15 Minutes	Break
16:00 – 18:00	120 Minutes	Evaluation:<br>Interviews with the country representatives

Day 3: 16th of January 2019

Time	Duration	Activity
8:30 – 9:15	45 minutes	Icebreaker & Reflection
9:15 – 10:15	60 Minutes	Plenary & Group work:<br>Sustainability and Fund raising
10:15 – 10:30	15 Minutes	Break
10:30 – 12:00	90 Minutes	Group Activity:<br>FIRO-B - The Fundamental Interpersonal Relations Orientation Behavioral instrument
12:00 – 13:00	60 Minutes	M&E<br>Reporting and the SHIRIM documentation
13:00 – 14:00	60 Minutes	Lunch
14:00 – 14:30	30 Minutes	Next steps
14:30 – 15:00	15 Minutes	Closing remarks

Supplement to: Meijers K, van der Kwaak A, Aqel I, et al. Using a rapid knowledge translation approach for better sexual and reproductive health and rights in Bangladesh, Burundi, Indonesia, and Jordan. *Glob Health Sci Pract*. 2022;10(2): e2100461.
https://doi.org/10.9745/GHSP-D-21-00461

SUPPLEMENT 5. COUNTRY KNOWLEDGE PLATFORM CASE STUDIES

Case Study Bangladesh

The knowledge platform[17] in Bangladesh is hosted by an organization that works on social and behavioral change communication and advocacy on SRHR issues.[18] The platform together with its members identified the lack of collaboration and the absence of knowledge on the consequences of child marriage (both physical and mental) at the field level due to lack of concentrated effort between the government, NGOs and CSOs as the main topic. These knowledge gaps as well as communication gaps resulted in limited knowledge sharing and translation into effective interventions to address CM.

In December 2017, the Ministry of Women and Children Affairs passed the controversial Marriage Restraint Act which stated 'if a marriage is solemnized in such manner and under such special circumstances as may be prescribed by rules in the best interests of the minor, at the directions of the court and with consent of the parents or the guardian of the minor, as the case may be, it shall not be deemed to be an offence under this Act'. This act has been subject to criticism from rights activists throughout the country and abroad, as it creates a loophole for parents to marry off their children legally. Besides, there is a growing number of interventions both by the Government and NGOs are addressing the issue of CM in Bangladesh. However, the country remains among the top ten countries in the world for CM. Therefore, the knowledge platform together with its members focused on addressing lack of collaboration and absence of knowledge throughout the learning sessions and action periods.

During the first action period, an information hub on the knowledge platform webpage to highlight projects that focus on CM, was created by the knowledge platform secretariat with support from the members of the CoP on CM. This was achieved through mapping the projects and disseminating an overview of research and interventions on CM in Bangladesh from 2010-2018.

During the second learning session, ten experts on CM were interviewed by team members of the knowledge platform and the results were published on the webpage to support the translation of tacit knowledge into explicit knowledge. Additionally, in collaboration with the members of the knowledge platform, a booklet titled 'Highlights of Initiatives Addressing Child Marriage in Bangladesh' (see figure 4) was developed by the knowledge platform to show the best practices for prevention of CM among actors in Bangladesh.[28] This activity highlighted the field experience from experts by translating tact knowledge to explicit knowledge.

The knowledge platform secretariat distributed the booklet among members of the platform and practitioners working on CM to help them learn about the work that other organizations do to prevent CM. During a knowledge fair, organized as an annual activity by the knowledge platform, the booklet and the knowledge collaboration between actors was presented and shared.

In the third action period, the knowledge platform together with the Child Marriage CoP members and the Royal Netherlands Embassy in Dhaka, organized a round-table discussion (see figure 5) to share the lessons learned in promoting the collaboration and agree on coordination among stakeholders, including the government of Bangladesh. Current strategies that effectively address CM in Bangladesh, lessons learned, challenges and follow-up action were discussed. During this meeting, the CRIM-KT methodology has shown to facilitate the identification of knowledge gaps and the translation from tacit knowledge of experts to explicit knowledge. The local stakeholders were impressed by the methodology and expressed their interest in participating again in such collaboration in addressing different SRHR issues. Practice changed after CRIM-KT was implemented. The collaboration between the CoP on CM, and government officials has intensified and facilitates evidence-informed working.

Participants mentioned as reasons for the successful change in bringing together stakeholders and strengthened coordinating efforts, that the approach provided structure to capitalize this experience in a systematic way and

enabled stakeholders to discuss which change ideas are feasible in terms of budget and time constraints, while providing space for experiencing new ways of working. Barriers were the varying degree of willingness of stakeholders to work together and the limited existing collaboration between NGOs and the government. Additionally, high staff turnover within participating organizations resulted in new stakeholders taking part in each learning session that needed to be updated.

Case Study Burundi

The Burundian knowledge platform[18] was hosted by an international non-governmental organization. The platform identified teenage pregnancies as their focus. The incidence of school-aged pregnancies in Burundi is alarming. Only little evidence was available on the topic of TP. Therefore knowledge generation was identified by the representatives from the knowledge platform, the National Reproductive Health Program of Burundi (PNSR) and the National Institute of Public Health as a key priority during the first learning session. Participants developed a change package which included a desk review as well as a stakeholder mapping and analysis. The mapping of stakeholders (see figure 6) working on teenage pregnancy was conducted by the representatives of the knowledge platform with technical support from Share-Net International (SNI). Furthermore, the knowledge platform initiated and coordinated a desk review of teenage pregnancy interventions in international literature performed by an intern at SNI. The desk review elaborated on known best interventions to reduce or prevent adolescent pregnancy in Burundi, Kenya, Ethiopia & the Demographic Republic of Congo. A reflection paper was written by the same intern on how the Joint Program Intervention Should or Might Improve Adolescent Pregnancy in Burundi. The Joint Program is a five-year partnership with the aim to improve SRHR of adolescents and young people in Burundi.

During the second action period, a joint broadcasting initiative in the form of a talk show on 12 radio stations was organized by the knowledge platform with support from the host organization to make the general public more aware of risk of teenage pregnancies. The knowledge platform coordinator and the National Reproductive Health Program director participated in the panel to respond to direct questions from the audience. This activity was financially supported by an external funder.

In the third action period, a round table (see figure 7) was organized by the knowledge platform representatives to reflect on improving mechanisms for monitoring teenage pregnancies in Burundi. During the round table, participants proposed to the National Reproductive Health Program representatives to conduct a thorough analysis of existing monitoring mechanisms of adolescent pregnancies in Burundi in order to identify concrete knowledge gaps and propose correcting measures. The report 'Analyzing the current mechanisms of monitoring adolescent pregnancies in Burundi' was drafted in French by a team encompassing representatives from the National Reproductive Health Program, the National Health Information Management System and the knowledge platform secretariat.[29] The report included recommendations on how the existing health information system of the government can improve the documentation of TP at the national level.

CRIM-KT in Burundi has proven to be a useful strategy to foster collaboration between stakeholders within a short timeframe with clear outputs in the form of different knowledge products. The participants mentioned, as a barrier for change, that focusing on knowledge translation strategies while there is no current data and existing literature available on TP in Burundi, made it challenging to improve policy and practice. The other barrier mentioned was the limited financial budget to organize and execute activities. Organizing the local learning sessions and action periods was relatively expensive compared to the other hubs, because of stipends paid to participants, price of venue rental, and facilitator fees.

In Burundi the local learning sessions and action periods could only be organized by the knowledge platform after receiving prior permission from the Burundian government. Furthermore, due to governmental regulations, the learning sessions had to be partly facilitated by the Program Manager of National Reproductive Health Program

(PNSR) even though this person was not present during the international learning sessions during which the representatives of Share-Net Burundi were trained on the methodology.

The second learning session was organized later than originally planned because the Burundian government suspended all workshops in May 2018 to prioritize the local elections. End of September 2018, the Burundian National Security Council decided to ban international NGOs for the consecutive three months. This ban also suspended operations of the host of Share-Net Burundi. This meant a delay in organizing both the second and the third learning session.

Case Study Jordan

The knowledge platform in Jordan is hosted by the Higher Population Council (HPC), a semi-governmental organization with the authority to direct national efforts on sustainable development, population issues, and reproductive health. The knowledge platform selected child marriage as their topic because sufficient evidence on the harmful consequences CM already existed and more collective efforts were needed to make sure that different actors, including the government, took collaborative action to implement policies and programs to address CM.

During the first local learning session, stakeholders decided to develop a national action plan to end CM.[26] The plan aimed to provide a general framework for limiting the marriage of individuals under the age of 18 and a manual of short and medium-term national interventions for the following five years. The action plan was formulated based on the results of a comprehensive study and a policy brief on CM in Jordan.[30]

The planning process consisted of several stages that took place across the different learning sessions and action periods, including four round table discussions with different actors. The action plan took into account the financial capabilities and human resources of the national implementing partners and its related programs. The final action plan was successfully presented by the Secretary General of the HPC to the Prime Minister, and endorsed by the cabinet. Furthermore, as a result of this whole CRIM-KT process, the legal age for marriage increased from 15 to 16 years. The platform held several meetings with potential donors to share the action plan for reducing CM in the country and the executive program for the years 2018-2022 to explore funding opportunities for the different activities included in the plan. With external funds, a workshop on the mechanisms for integrating CM in the plans of civil society institutions and societies was held.

During the second and third local learning session, a national multi-stakeholder partnership was established by the King Hussein Foundation. During the following action periods, the partnership raised their own funds and implemented joint activities. In total seven stakeholders participated in conducting a research to understand the economic and social drivers that influence CM in Jordan. An awareness training on CM was conducted by two stakeholders at hospitals for 100 participants. A multi-stakeholder project targeting Syrian refugee girls at schools (aged between 14 and 16) and their families using health and socioeconomic approach to reduce CM was developed and externally funded. Two partners conducted an awareness campaign at 60 schools around CM.

Knowledge experts and participants deemed as reasons for the successful change in Jordan, the involvement and leadership of HPC as a semi-governmental organization which led to more participation from the public sector and NGOs. Additionally, multi-stakeholder approaches set-up during the local learning sessions to address CM were successful. The host organization produced a detailed study and a policy brief on CM together with the Jordanian knowledge platform prior to the start of the CRIM-KT approach. in the recommendations from the policy brief supported evidence-based decision making and led to the approval of the study and the national action plan by the Prime Minister and cabinet.

Barriers identified by the participants to implement the approach were that not all stakeholders had the same willingness to put efforts in addressing CM. Most prominently is the limited available funding from governmental

and non-governmental entities to implement the national action plan. Lastly, there was a lack participation of the private sector.

Case Study Indonesia

An international non-governmental organization, which was part of a large five year program on reducing child marriage, teenage pregnancy and female genital mutilation (FGM) in Indonesia[20] implemented the rapid improvement model. As theme, they identified the harmful interpretation of the *merarik* culture (elopement) that supports 'kidnapping' of young girls which influences the occurrence of CM and a high divorce rate.[31]

In preventing CM in West Lombok, each stakeholder tends to work independently (government institutions and local CSOs), and not involving religious and traditional leaders. Through the CRIM-KT approach in West Lombok, the leading INGO together with another member of the alliance gathered all stakeholders/gatekeepers. Increased collaboration of these various stakeholders was facilitated through several learning sessions. During the first learning session participants mentioned as reasons to select this topic, the available evidence on harmful consequences of CM, the alignment with local government priority and the strong linkage with interests of the existing multi-stakeholder district working group. The misinterpretation of the merarik culture was identified by the stakeholders as a root cause. The first change package focused on increasing understanding around the merarik culture and engaging stakeholders and conducting a needs assessment among stakeholders. Furthermore, the existing district working group was strengthened by acquiring co-funding from government to organize meetings and by planning and organizing more structural and systematic meetings with agenda setting focusing on improving policy and practice in relation to CM in West-Lombok. This resulted in a Memorandum of Understanding (MoU) among stakeholders to address CM because of elopement. Customary laws that have only been conveyed by mouth to mouth tend to experience a shift in meaning. In order to return the customary merarik law to its original meaning, a written document was needed. That is why, during the second action period, the involved stakeholders agreed the change package would focus on the development and launching of a technical guideline to clarify and bring back the authentic concept and process of merarik.[27] Subsequently, village heads in Lombok received training from religious leaders on the guideline to be able to refuse to give permission for CM. The traditional leaders facilitating the trainings were supported by the District Office of Population Control, Family Planning, Women's Empowerment and Child Protection and the INGO. The training of village heads on the guideline was integrated by the leading INGO in the 5-year program to address CM, teenage pregnancy and FGM. During the third action period a multi-stakeholder partnership was set-up to develop a syllabus for training on merarik guideline and engaging stakeholders to adopt the guidelines through advocacy efforts. The implementation of the guideline was monitored and documented by the INGO that implemented the rapid improvement model. In 2019, the local guideline on the merarik cultural practice was integrated in the existing Local Regulation of Child Marriage Prevention issued by the parliament in West Lombok which can be considered a long term sustainable change. Furthermore, throughout the process three off- and online newspaper articles regarding the merarik guideline were published. The Department of Women Empowerment and Family Planning of West Lombok allocated budget to expand the coverage of the training in the villages outside the 5-year program.

Participants from Indonesia and knowledge experts gave several reasons for successful change in Indonesia. These include the alignment with stakeholder priorities, support from local leaders, availability of the already existing multi-stakeholder district working group, the embedding in a large 5-year program with strong partners, input from stakeholders about the feasibility of the project, and the implementation of the model at local instead of national level as the scale is smaller and more feasible. Participants valued the approach for its encouragement to prioritize activities, think about next steps and consider the feasibility of achieving change in each action period. Also, the intensity of the approach, in terms of number of action periods in short time, and the specific focus were said to be important factors. Elements that hampered implementation of the rapid improvement model were language barriers

to translate English concepts and exercises of the international learning sessions to Bahasa for the local learning sessions, the earthquake in Lombok in August 2018 and the staff turnover in organizations.
